# Supplementary material for: PAM-flexible Engineered FnCas9 variants for robust and ultra-precise genome editing and diagnostics
Source: Nat Commun. 2024 Jun 28;15:5471. doi: 10.1038/s41467-024-49233-w (PMC11213958; doi:10.1038/s41467-024-49233-w)
Supplement: Supplementary file 1 — Supplementary Information [file 41467_2024_49233_MOESM1_ESM.pdf]

Supplementary Information

PAM-flexible Engineered FnCas9 variants for robust and ultra-precise genome editing and diagnostics

Sundaram Acharya[1,2,#], Asgar Hussain Ansari[1,2], Prosad Kumar Das[1], Seiichi Hirano[3], Meghali Aich[1,2], Riya Rauthan[1,2], Sudipta Mahato[4,5], Savitri Maddileti[4], Sajal Sarkar[1,2], Manoj Kumar[1,2], Rhythm Phutela[1,2], Sneha Gulati[1], Abdul Rahman[1], Arushi Goel[1,2], C. Afzal[1], Deepanjan Paul[1],Trupti Agrawal[4,5], Vinay Kumar Pulimamidi[4,6], Subhadra Jalali[7], Hiroshi Nishimasu[8,9,10], Indumathi Mariappan[4], Osamu Nureki[3], Souvik Maiti[1,2], Debojyoti Chakraborty[1,2,#]

[1]CSIR-Institute of Genomics & Integrative Biology, Mathura Road, New Delhi- 110025, India

[2]Academy of Scientific & Innovative Research (AcSIR), Ghaziabad, 201002, India

[3]Department of Biological Sciences, Graduate School of Science, The University of Tokyo, 7-3-1 Hongo, Bunkyo-ku, Tokyo 113-0033, Japan

[4]Center for Ocular Regeneration, Prof. Brien Holden Eye Research Centre, Hyderabad Eye Research Foundation, LV Prasad Eye Institute, Hyderabad-500034, Telangana, India

[5]Manipal Academy of Higher Education, Manipal University, India

[6]Schepens Eye Research Institute, Massachusetts Eye and Ear, Harvard Medical School, Boston, MA 02114, USA.

[7]Srimati Kannuri Santhamma Centre for vitreoretinal diseases, Anant Bajaj Retina Institute, Kallam Anji Reddy Campus, L V Prasad Eye Institute, Hyderabad, Telangana, India.

[8]Department of Chemistry and Biotechnology, Graduate School of Engineering, The University of Tokyo, 7-3-1 Hongo, Bunkyo-ku, Tokyo 113-8656, Japan

[9]Research Center for Advanced Science and Technology, The University of Tokyo, 4-6-1 Komaba, Meguro-ku, Tokyo 153-8904, Japan

[10]Inamori Research Institute for Science, 620 Suiginya-cho, Shimogyo-ku, Kyoto 600-8411, Japan

#Correspondence:

acharyasundaram.ac@gmail.com (S.A.); debojyoti.chakraborty@igib.in (D.C.)

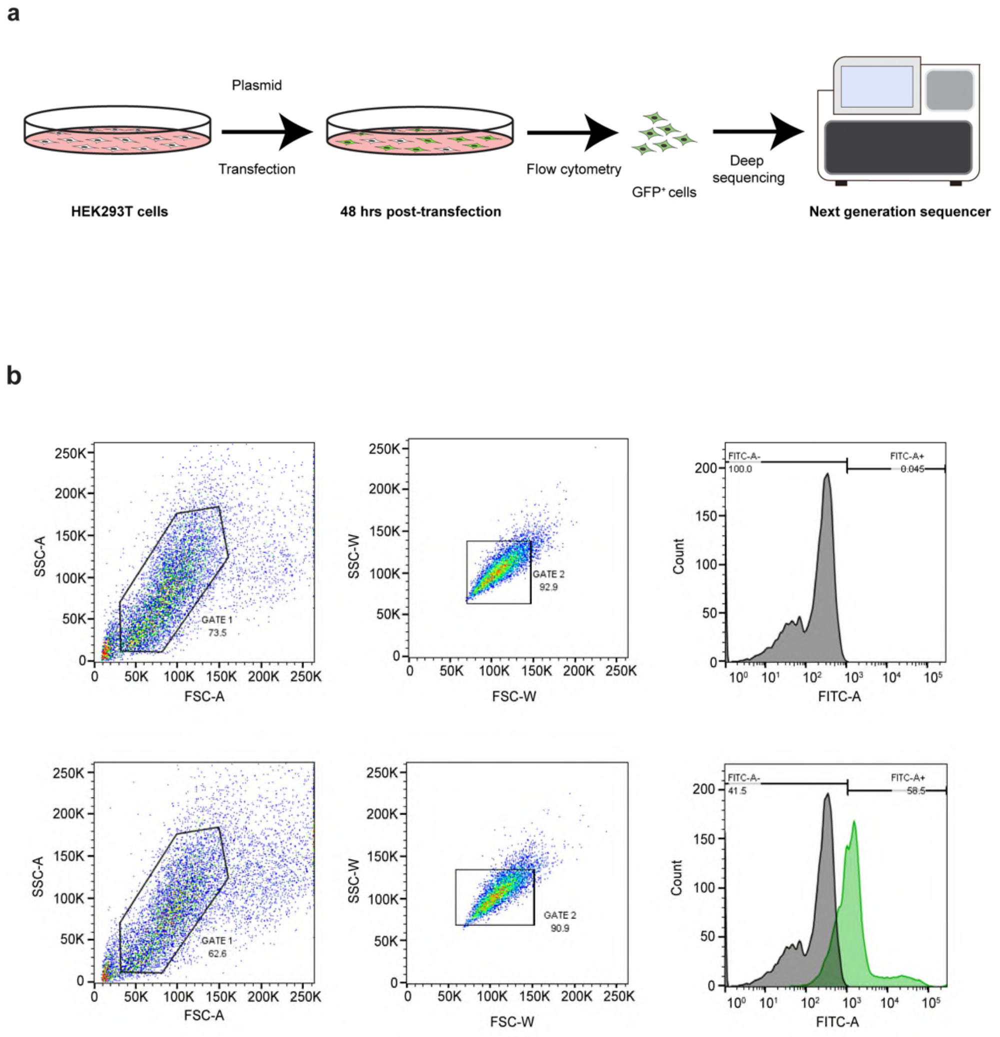

Supplementary Fig. 1: Experimental pipeline of cellular editing assays.

a Schematic showing the experimental pipeline used for genome editing efficiency evaluation. **b** Representative gating strategy for flow cytometry experiments. GATE 1 to include population without cell debris, GATE 2 illustrates single cell gating and histogram gating identifies FITC-A- (GFP negative) and FITC-A+ (GFP positive) fluorescence event distribution in control (upper) and test samples (lower).

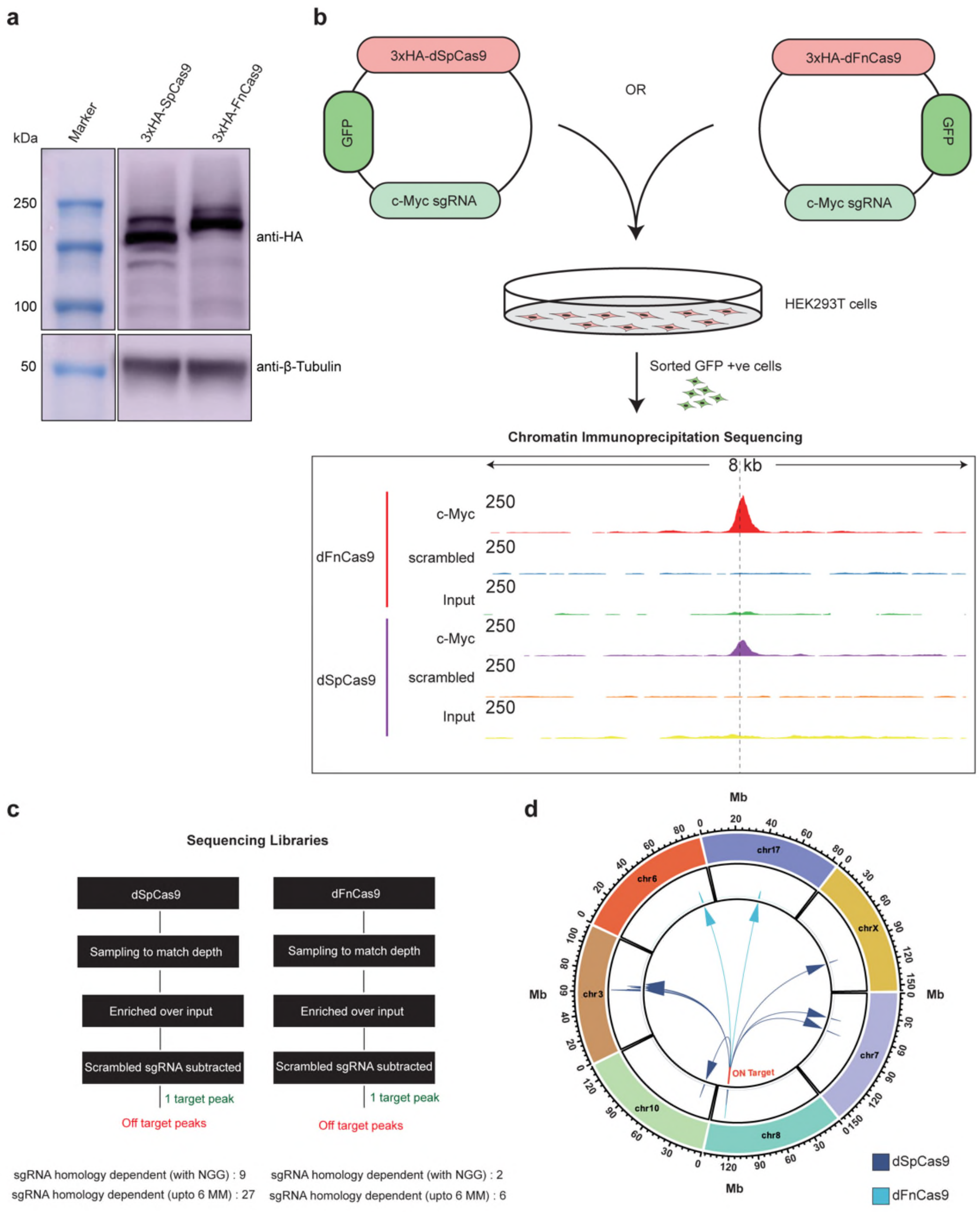

Supplementary Fig. 2: Genome-wide binding sites of dSpCas9 and dFnCas9 in HEK293T cells.

a Western blot analysis showing the expression of HA-tagged SpCas9 and FnCas9 from the whole cell lysates transfected with the respective expression constructs, and probed with α-HA. Tubulin was used as a loading control.

b Schematic showing the ChIP-seq assay for targeting *c-Myc* locus by 3xHA-dSpCas9 and 3xHA-dFnCas9. ChIP-seq signal intensity of dSpCas9 and dFnCas9 w.r.t. input sample and scrambled sgRNA transfection at the intended on-target region is shown.

c Peak calling pipeline showing the off-target sites of 3xHA-dSpCas9 and 3xHA-

dFnCas9 ChIP-seq assay on *c-Myc* locus. **d** Circos plot showing the sgRNA homology dependent off-target peaks (calculated up to 6 mismatches, MM) with 5'-CGG-3' PAM for dSpCas9 and dFnCas9. Chromosomes are arranged radially and shown in megabase (Mb) for which sequence reads were captured for both the dCas9s. On-target peaks for both the dCas9s are shown and marked in red. The Source Data are provided in the source data file.

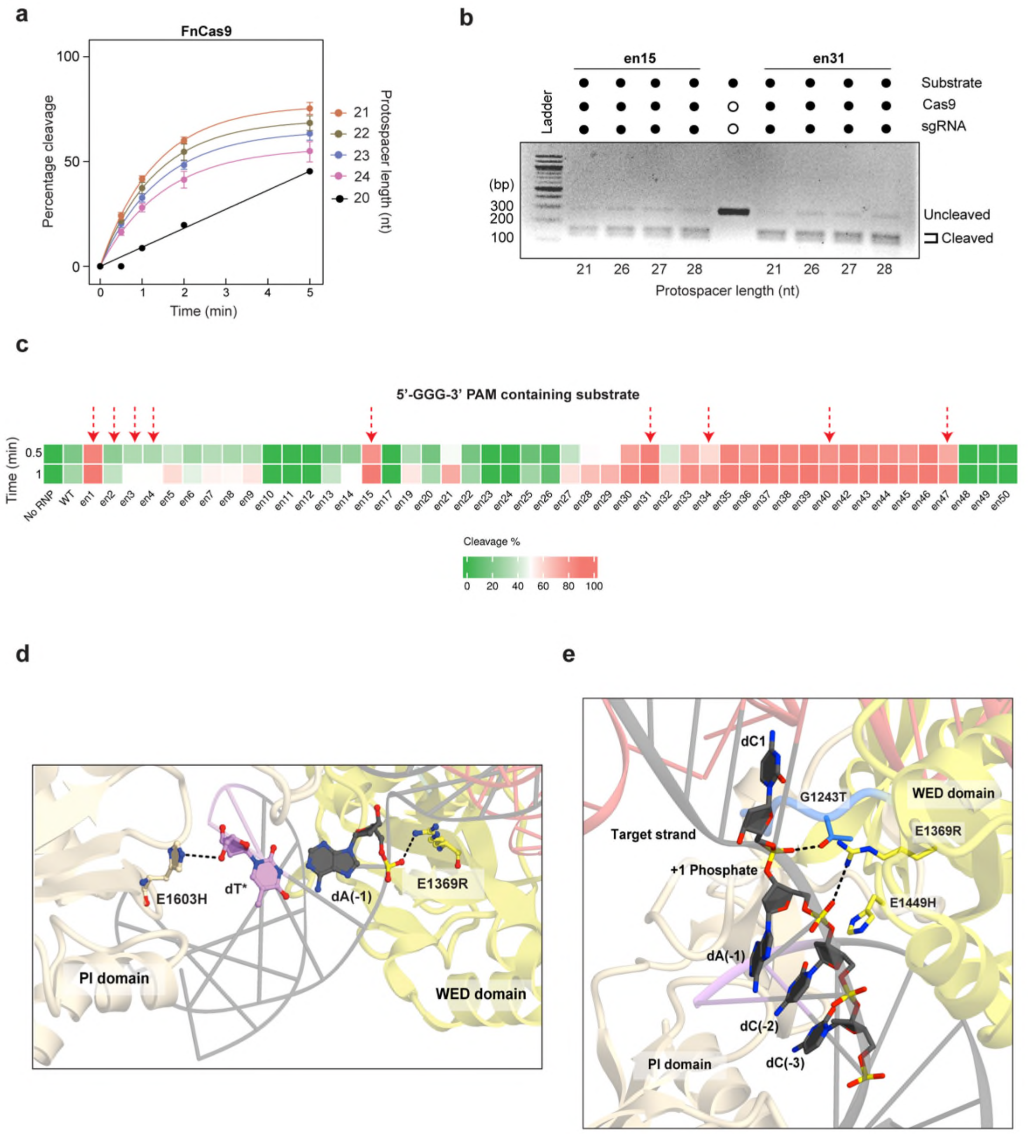

Supplementary Fig. 3: Screening of FnCas9 variants for enhanced cleavage activity.

a Fitted line plot showing the rates of *in vitro* DNA cleavage of FnCas9 (expressed in percentage, Y-axis) as a function of time (expressed in minutes, on X-axis) upon programmed with variable length of sgRNAs having protospacer length ranging from 20 to 24 nucleotides (nt). Error bars represent mean ± SD of n=3 independent experiments. **b** Agarose gel image of the *in vitro* cleavage assay showing the DNA cleavage efficiency of en15 and en31 upon programmed with variable length of sgRNAs having protospacer length ranging from 21, 26 to 28 nucleotides (nt) (n=1). 25 nM DNA substrate was incubated with 250 nM RNP for 15 minutes. Reaction was

quenched and treated with Proteinase K before running on 2% agarose gel. Solid dot and open dot represent the presence and absence of components in the assay respectively. **c** Heat map showing the *in vitro* cleavage screening assay of FnCas9 and enFnCas9 variants using 5’-GGG-3’ PAM containing DNA substrate expressed as cleavage percentage for 0.5 and 1 minutes. Red dotted arrows indicate the subset of nine enFnCas9 variants selected for studying enzymatic activity as a function of time (n=1). **d** Structural model showing the interactions of en1 (E1369R) and en15 (E1603H) mutations with the PAM duplex. **e** Structural model showing the interactions of en31 mutations (G1243T/E1369R/E1603H) with PLL loop and PAM duplex. Source Data are provided in the source data file.

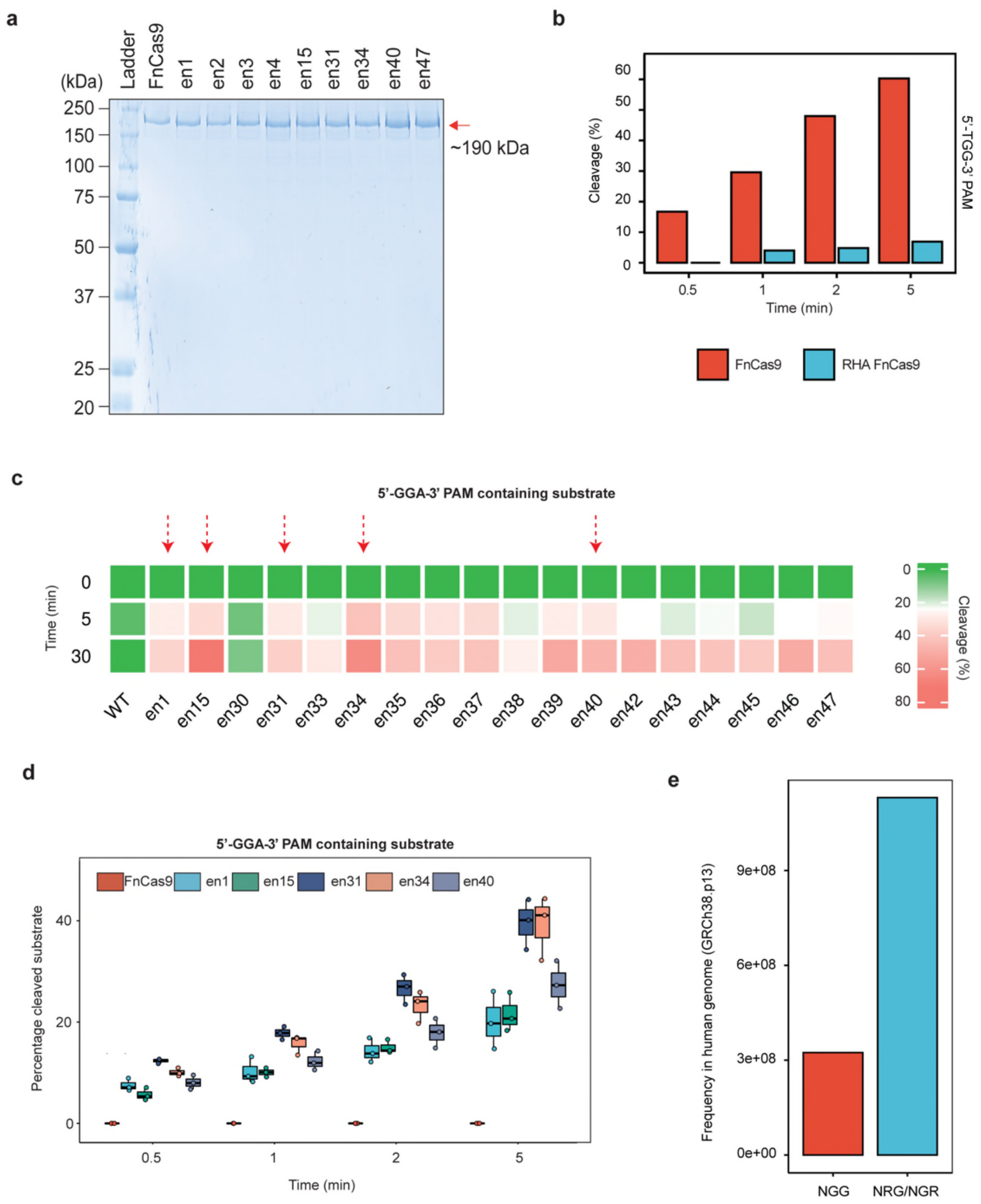

Supplementary Fig. 4: Characterization of altered PAM specificities by enFnCas9 variants.

a Coomassie stained SDS-PAGE gel showing purified FnCas9 and a subset of enFnCas9 protein variants used in the study. **b** Comparative evaluation of *in vitro* cleavage activity between FnCas9 and RHA FnCas9 on 5'-TGG-3' PAM containing substrates represented by percentage cleavage (y-axis) as a function of time (x-axis) (n=1). **c** Heat map showing the *in vitro* cleavage screening assay of FnCas9 and a subset of enFnCas9 variants using 5'-GGA-3' PAM containing DNA substrate expressed as cleavage percentage for 5 and 30 minutes (n=1). Red dotted arrows

indicate the subset of five enFnCas9 variants selected for studying enzymatic activity as a function of time. **d** Box plot showing in vitro cleavage assay using 5’-GGA-3’ PAM containing DNA substrate expressed as cleavage percentage (y-axis) as a function of time (x-axis). Error bars represent mean ± SD of n=3 independent experiments. **e** Bar plot showing the calculated frequency of 5’-NGG-3’ and 5’-NRG/NGR-3’ PAM in the human genome (GRCh38.p13). Source Data are provided in the source data file.

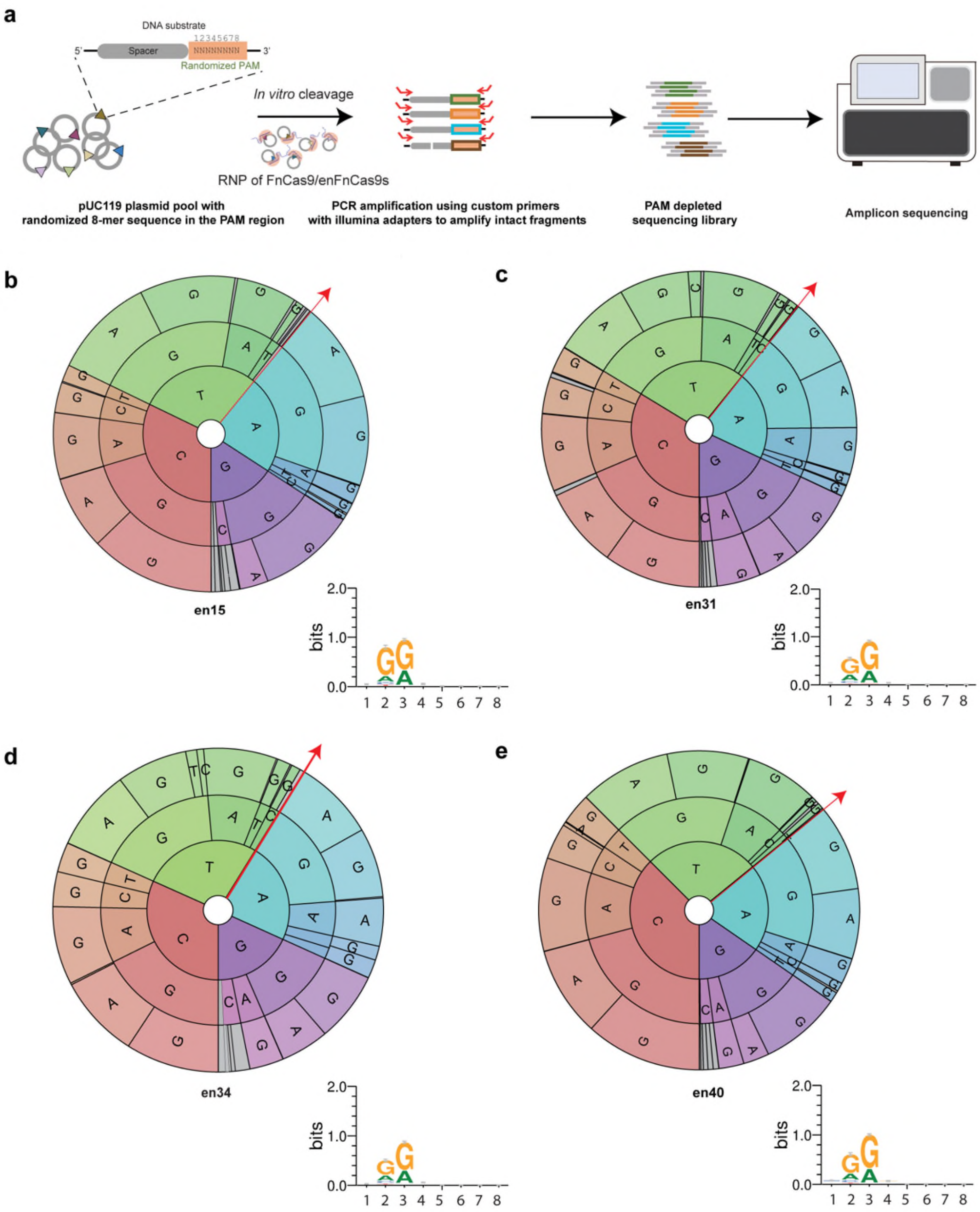

Supplementary Fig. 5: Characterization of comprehensive PAM profiles of enFnCas9 variants.

a Schematic representation showing the pipeline of PAM discovery assay. Main steps in the assay are highlighted. **b** The PAM wheels and sequence logos showing the results obtained from PAM discovery assay for FnCas9, en1, en15, en31, en34 and en40. Individual sections of the pie charts in the PAM wheels with ≤ 2% depletion enrichment are shown in gray. Bases from the inner to the outer circle in the PAM wheels map the PAM reads away from the target region in the 5’ to 3’ direction as

shown by red arrows. Source Data are available at Figshare, 10.6084/m9.figshare.25827652.

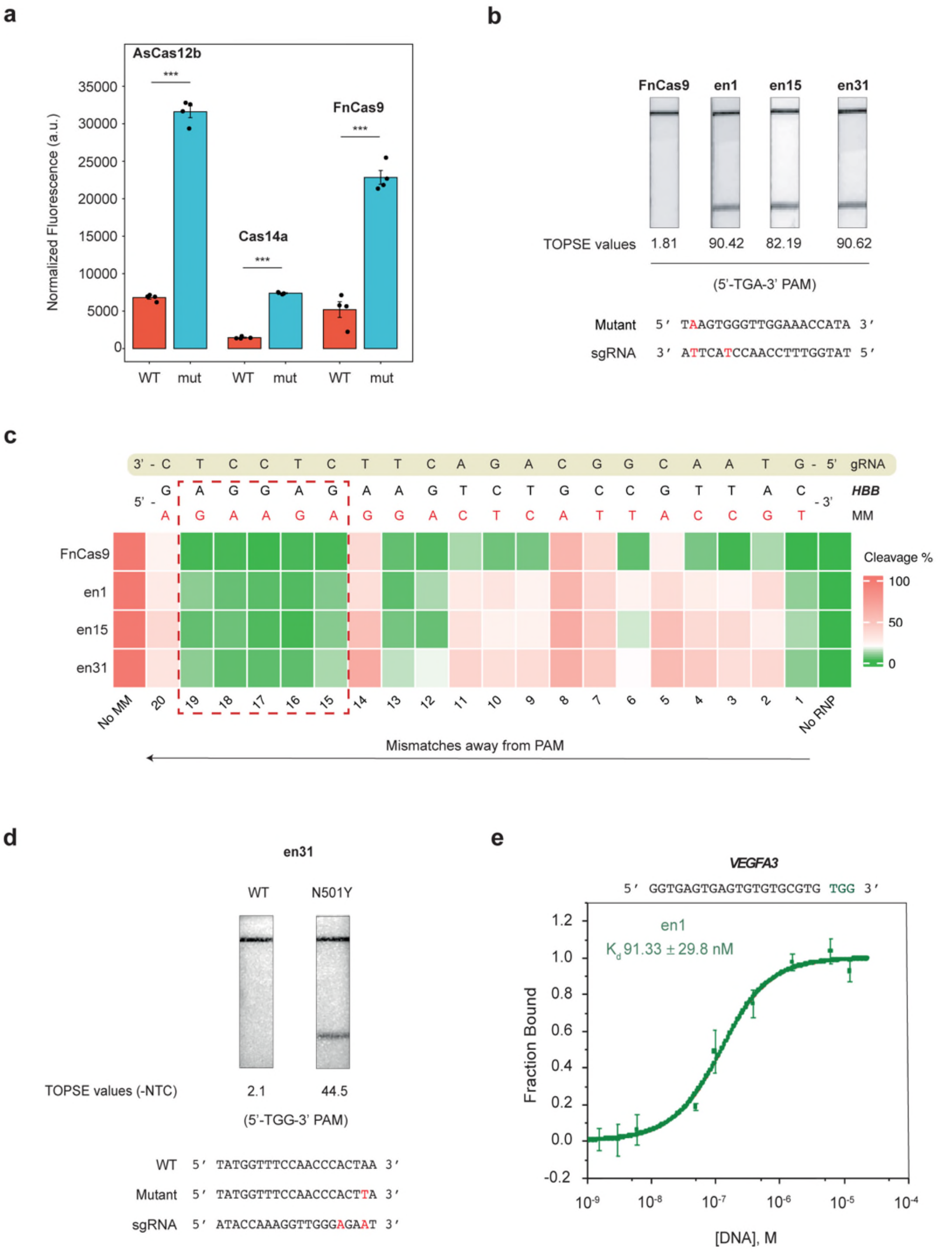

Supplementary Fig. 6: Characterization of enFnCas9 variants for mismatch sensitivity and its potential in CRISPRDx platforms.

a Bar plot showing the discrimination of a single mismatched substrate by dFnCas9, AaCas12b and Cas14a1 using fluorescence-based assays (affinity-based for FnCas9 and trans-cleavage for AaCas12b and Cas14a1). Error bars represent mean ± SEM of n=4 independent experiments. Unpaired two-tailed Student's *t*-test was applied and p-values are represented for *** <0.001. **b** Representative image showing the outcome of lateral flow assay (LFA) on a 5'-TGA-3' PAM containing substrate for FnCas9, en1, en15 and en31 (n=3, independent experiments). The target sequence (SARS-Cov2 N501Y, mutant) and the sgRNA spacer sequence (PAM-proximal 2nd/6th mismatches,

when counting away from PAM are in red. One mismatch w.r.t. mutant substrate) are shown. The target mutation is highlighted in red. Corresponding TOPSE values are given at the bottom. **c** Heat map showing the *in vitro* cleavage assay outcome (expressed cleavage %) of FnCas9, en1, en15 and en31 on *HBB* and its mutant substrates (n=1). Each substrate is carrying a single mutation (position is indicated by counting away from PAM) across the target length. Mismatch containing base is indicated in red while other unaltered bases are shown in black. 25 nM of DNA substrates were incubated with respective 100 nM RNPs for 15 min at 37°C. Reaction was quenched and treated with Proteinase K before running on 2% agarose gel. Dotted box shown in red represents the PAM-distal sites. The samples were derived from the same experiment and that agarose gels were processed in parallel. **d** Representative image showing the outcome of lateral flow assay (LFA) for SARS-Cov2 N501Y (with a 5'-TGG-3' PAM) mutation detection by en31 using RAY (n=3, independent experiments). WT and N501Y target sequences are shown. The N501Y mutation is highlighted in red. The sgRNA spacer sequence is shown and mismatches (PAM-distal $16^{th}/19^{th}$, when counting away from PAM) are shown in red. Corresponding TOPSE values are given at the bottom. **e** MST analysis showing the binding affinity of en1 on VEGFA3 substrate DNA. Data is represented as a fraction bound RNP (y-axis) with respect to purified DNA substrate (Molar units M, x-axis). Error bars represent mean ± SD of n=3 independent experiments. Source Data are provided in the source data file.

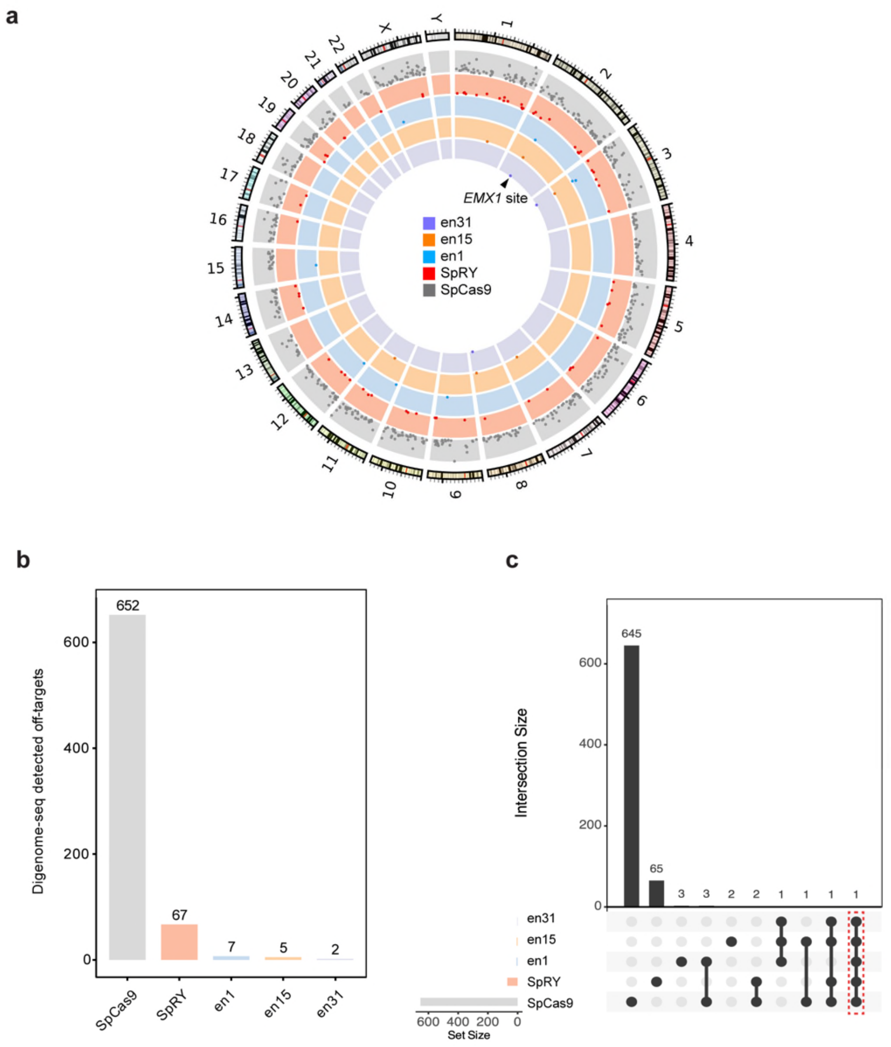

Supplementary Fig. 7: Comparative genome-wide off-targeting profile of SpCas9, SpRY and enFnCas9 variants in human cells.

a Circos plot showing the comparative off-targeting profile by SpCas9 and SpRY programmed with 20-nt spacer containing sgRNA, and en1, en15 and en31 programmed with 21-nt spacer containing sgRNA against *EMX1* locus in HEK293T cells as captured by Digenome-seq. The scatter dots indicating the on-target site (marked by arrow head) and off-target sites (n=1). Each concentric circle corresponds to each Cas9 variant as labeled in the figure. **b** Bar plot showing the number of off-target sites plotted on the Y-axis from Digenome-seq assay for SpCas9, SpRY, en1, en15 and en31 at EMX1 locus in HEK293T cells. **c** UpSet plot showing the hits identified in the Digenome-seq at *EMX1* locus across Cas9 variants. The on-target is outlined with a dotted red rectangle. Source Data are provided in the source data file.

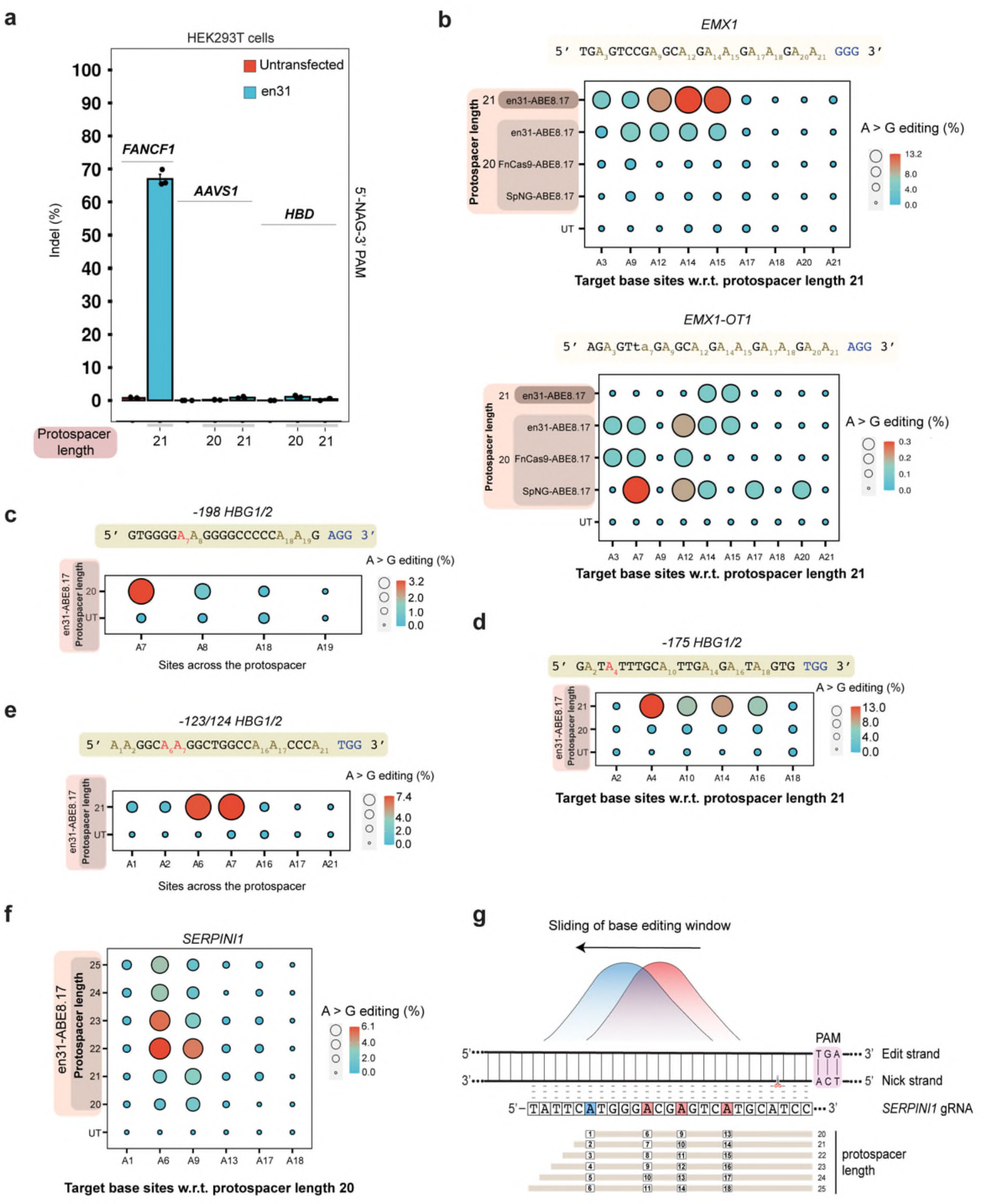

Supplementary Fig. 8: Characterization of enFnCas9-based nuclease and base editing systems in human cells.

a Bar plot showing the indel events (%) plotted on the Y-axis as obtained from amplicon sequencing upon targeting 5’-NAG-3’ PAM containing *FANCF1* site 2, *AAVS1* and *HBD* loci by en31 with sgRNA containing either 20-nt spacer (g20) or 21-nt spacer (g21) in HEK293T cells. Untransfected cells were used as control. Error bars represent mean ± SEM of n=3 independent biological replicates with individual values shown as

dots. **b** Ballon plot showing the A to G editing events (%) as obtained from amplicon sequencing upon targeting *EMX1* locus (top) alongside its validated off-target, EMX1-OT1 (below) by FnCas9-ABEmax8.17d, en31-ABEmax8.17d, and SpNG-ABEmax8.17d with sgRNAs containing either 20-nt spacer (g20) or 21-nt spacer (g21) in HEK293T cells. Target bases (As) for adenine base editing are on the X-axis and numbered w.r.t. g21. Mismatches on the protospacer are shown in lowercase. PAM is highlighted in blue. Values represent the mean of n=3 independent biological replicates. **c, d, e** Ballon plots showing the A to G editing events (%) as obtained from amplicon sequencing upon targeting the *-198*, *-175*, and *-123/124* sites of *HBG1/2* promoter respectively, by en31-ABEmax8.17d in HEK293T cells. Target bases (As) for adenine base editing are numbered on the X-axis with the target site indicated in red and the bystander site in beige. PAM is highlighted in blue. Values represent the mean of n=3 independent biological replicates. **f** Ballon plot showing the modulation of base editing window by en31-ABEmax8.17d expressed in percentage of A to G editing plotted on Y-axis using sgRNAs with extended and super-extended spacers (g20 to g25) as obtained from amplicon sequencing upon targeting *SERPINI1* locus against 5’-TGA-3’ PAM in HEK293T cells. Target bases (As) for adenine base editing are on the X-axis and numbered w.r.t. g20. Values represent the mean of n=3 independent biological replicates. **g** Schematic showing the sliding of the base editing window from primary window (shown in red) to secondary window (shown in blue) by extended (x-) or super-extended (sx-) gRNAs, and indicated by higher efficiency of A to G editing on *SERPINI1* locus with 5’-TGA-3’ PAM. Source Data are provided in the source data file.

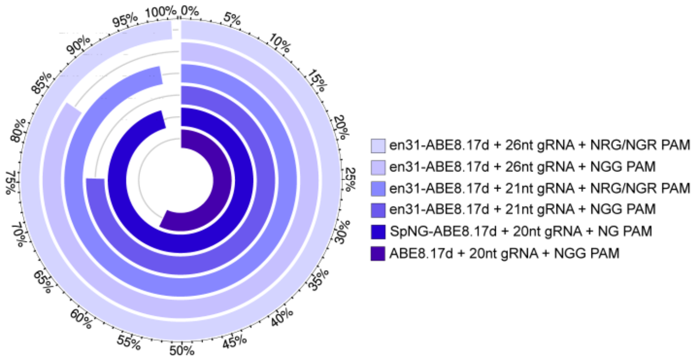

Supplementary Fig. 9: Human pathogenic SNPs targetable by en31-ABEmax8.17d and canonical ABE8s.

Circular bar plot showing the theoretically targetable pathogenic SNPs (expressed in percentage) as identified from the ClinVar for Sp-ABEmax8.17d (5'-NGG-3' PAM), SpNG-ABEmax8.17d (5'-NG-3' PAM) and en31ABEmax8.17d (5'-NGG/NRG/NGR-3' PAMs) with respective gRNAs of specified protospacer lengths. Source Data are provided in the source data file.

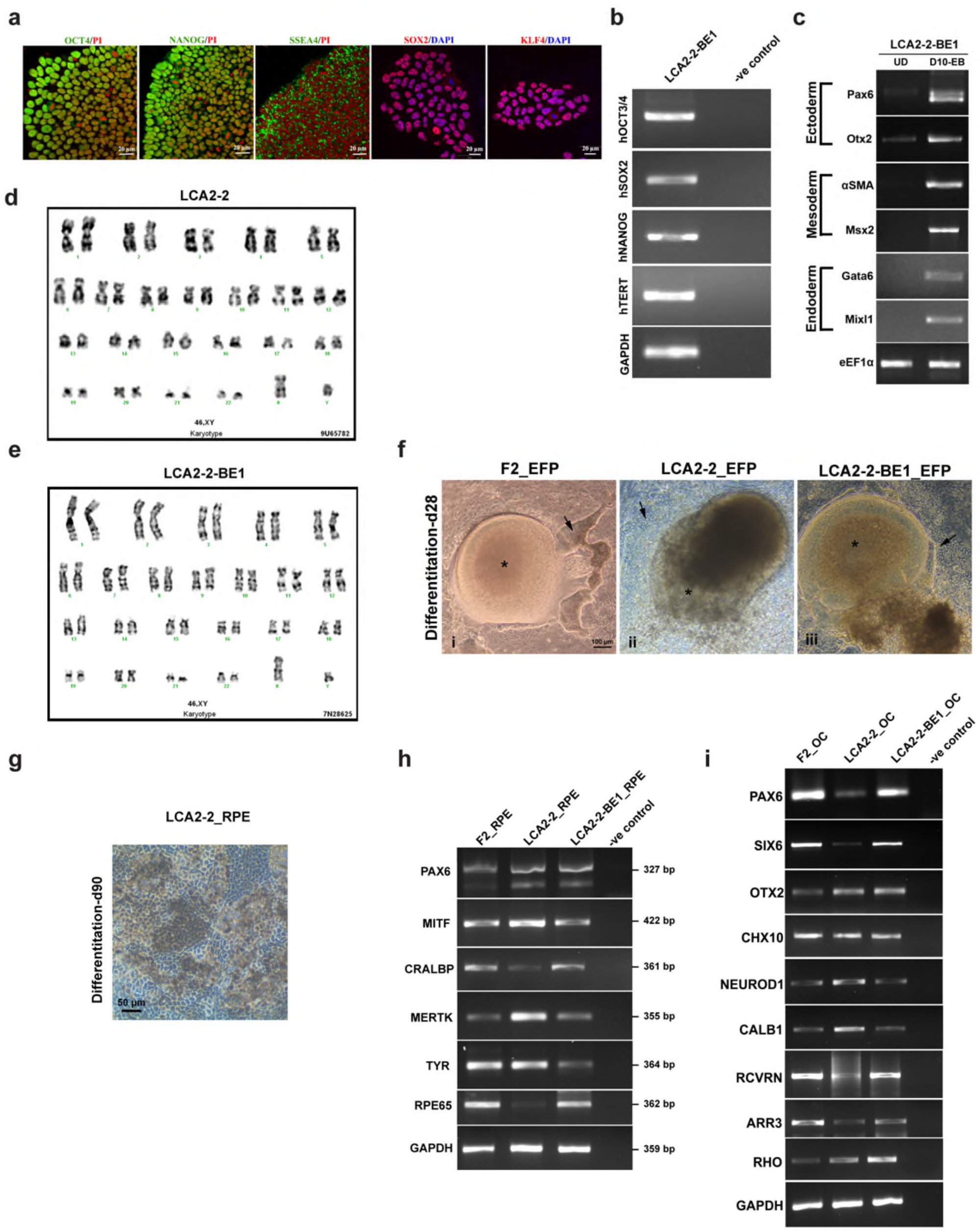

Supplementary Fig. 10: Characterization of adenine base edited and mutation corrected LCA2 patient-specific iPSC line (LCA2-2-BE1) and its retinal progenies.

a Representative images of iPSC colonies immunostained for the pluripotency markers (OCT4, NANOG and SSEA4 (in green), Sox2 and Klf4 (in red) and are shown as merged images with the counterstains, PI (in red) or DAPI (in blue) to mark all cell

nuclei (n=2 @ passage 10, 12). Scale bar: 20 µm. **b** Representative agarose gel images showing the semiquantitative RT-PCR results (n=2) for the expression of stemness markers (*hOCT4*, *hSOX2*, hNANOG and *hTERT*) in the mutation corrected patient-specific iPSC line, LCA2-2-BE1. GAPDH was used as the internal loading control. **c** Representative agarose gel images showing the semi-quantitative RT-PCR results (n=2) for the expression of trilineage markers namely; *PAX6, OTX2* (ectoderm); *α-SMA*, *MSX2* (mesoderm); *GATA6*, *MIXL1* (endoderm) in day 10 embryoid bodies (EB) derived from the mutation corrected patient-specific iPSC line, LCA2-2-BE1. Undifferentiated iPSCs (UD) were used as a control for trilineage markers. *eEF1α* was used as the internal loading control. **d, e** Representative karyotypes of the patient-specific iPSC line, LCA2-2 and the mutation corrected, the patient-specific iPSC clone 1, LCA2-2-BE1 at passage 12 showing a normal male karyotype. n=20 metaphase spreads were analysed. **f** Representative morphology of eye field primordial (EFP) clusters derived from healthy control iPSC line (F2), patient-specific mutant iPSC line (LCA2-2) and the mutation corrected patient-specific iPSC line (LCA2-2-BE1) at 3-4 weeks of retinal lineage differentiation (n=3). Asterisk (*) marks the central neuro-retinal island and black arrows indicate the migrating retinal pigmented epithelium. Scale bars: 100 µm. **g** Phase contrast image of the patient-specific iPSC (LCA2-2)-derived RPE cultures at differentiation day 90 (d90). Note the delayed appearance of pigmentation in mature RPE cells (n=3). **h** Representative agarose gel images showing the expression of RPE-specific gene transcripts such as *PAX6*, *MITF*, *CRALBP*, *MERTK*, *TYR* and *RPE65*, as analysed by semi-quantitative RT-PCR on day 45 of iPSC-RPE cells. GAPDH was used as an internal loading control (n=2). **i** Representative agarose gel images showing the expression of neuro-retina-specific transcripts such as *PAX6, SIX6, OTX2, CHX10, NEUROD1, CALB1, RCVRN, ARR3* and *RHO,* as analyzed by semi-quantitative RT-PCR in d35 retinal organoids (n=2) derived from the healthy control iPSC line, (F2-OC); the patient-specific iPSC line, (LCA2-2-OC); and the mutation corrected patient-specific iPSC line, (LCA2-2-BE-OC). *GAPDH* was used as the internal loading control.

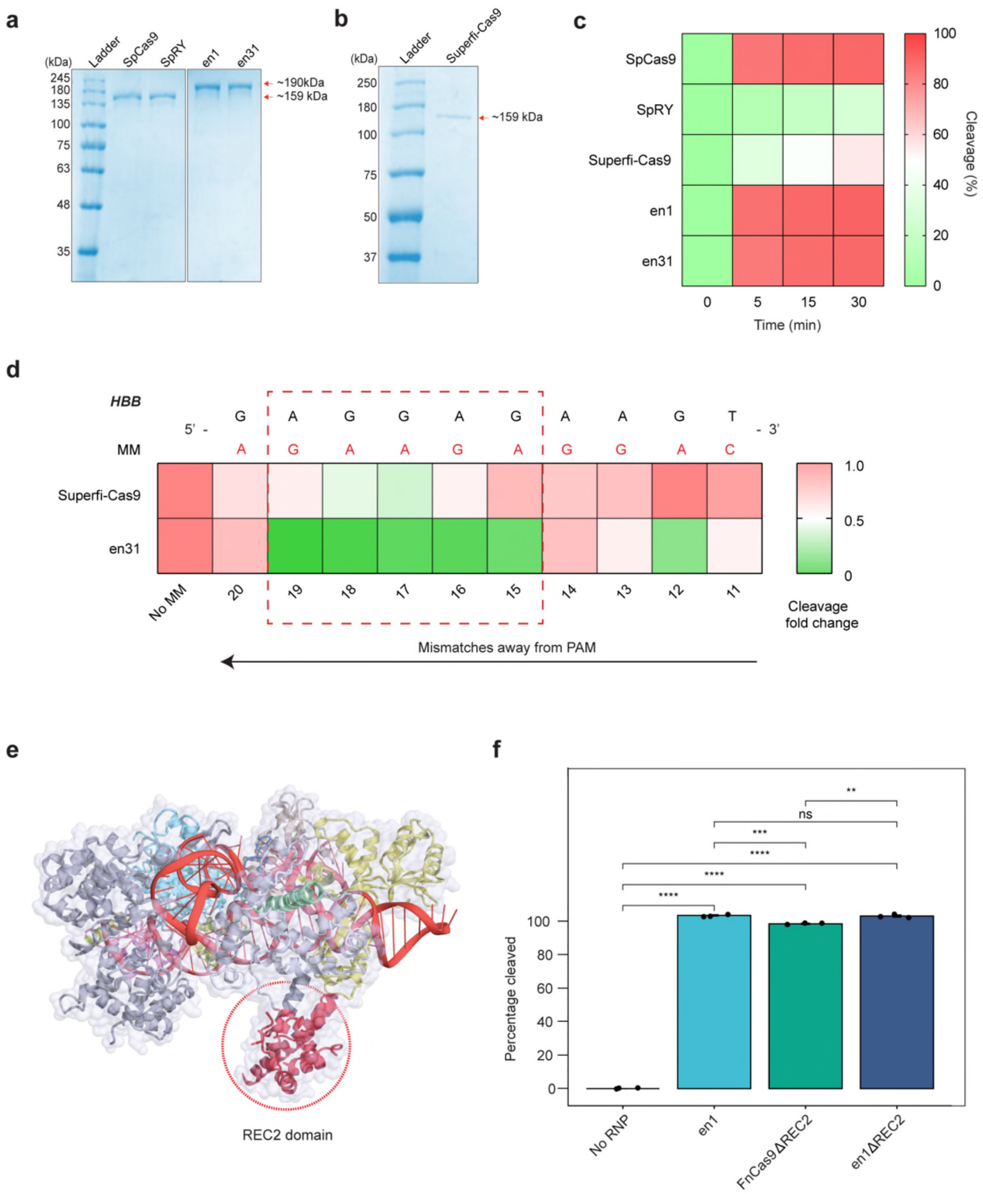

Supplementary Fig. 11: Comparative cleavage activity and specificity between SpCas9, SpRY, Superfi-Cas9 and enFnCas9 variants, and development of mini-enFnCas9 variant.

a, b Coomassie stained SDS-PAGE gel images of purified proteins. **c** Heat map showing the comparative *in vitro* DNA cleavage rate between SpCas9, SpRY, Superfi-Cas9, en1, en15 and en31 expressed as cleavage percentage for 0, 5, 15 and 30 minutes. Values represent the mean ± SEM of n=3 independent biological replicates. **d** Heat map showing the *in vitro* cleavage-based mismatch (MM) walking assay

outcome between Superfi-Cas9 and en31 on *HBB* and its mutant substrates, each carrying a single mismatch. The reaction was quenched and treated with Proteinase K before running on 2% agarose gel. Mismatches are indicated in red and counted away from PAM as indicated on the x-axis. The dotted box shown in red represents PAM-distal sites showing minimum mismatch tolerance. Data is shown as a cleavage fold change, normalized to *HBB* substrate (No MM) (n=1). **e** Crystal structure of FnCas9 in complex with DNA and RNA (PDB: 52BO) is shown in ribbon model with space-fill overlay. The truncated REC2 domain (ΔS112-A297) is highlighted in red and marked by a red-dotted circle. **f** Bar plot showing *in vitro* cleavage efficiency of en1, FnCas9ΔREC2 and en1ΔREC2 on 5'-GGG-3' PAM containing DNA substrate. The 5 nM DNA substrate was incubated with 100 nM RNPs for 1 hr at 37°C. Error bars represent mean ± SD of n=3 independent experiments. Unpaired two-tailed Student's *t*-test was applied and p-values are represented for ** ≤0.01, *** ≤0.001. Source Data are provided in the source data file.

Source data: Uncropped gel for Supplementary Fig. 2a

Supplementary Figure 2a

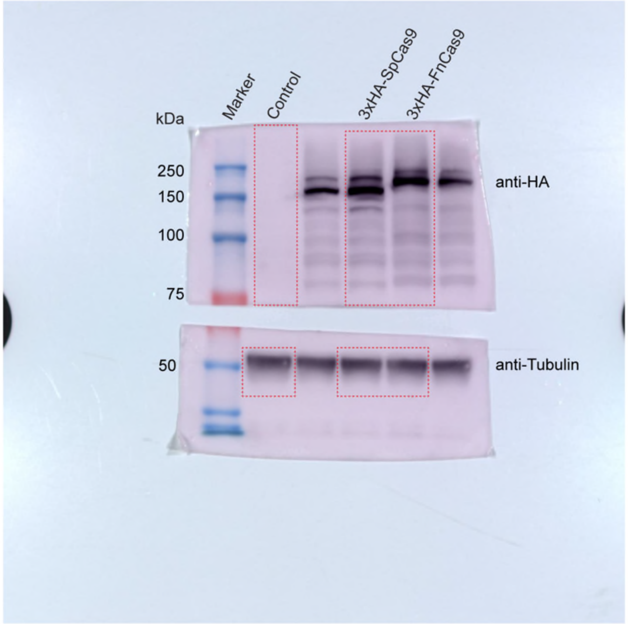

Source data: Uncropped gel for Supplementary Fig. 3a-c

Supplementary Figure 3a

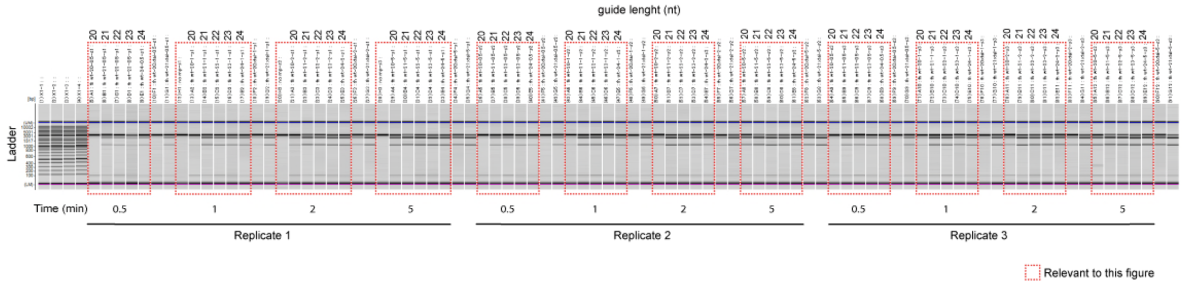

Supplementary Figure 3b

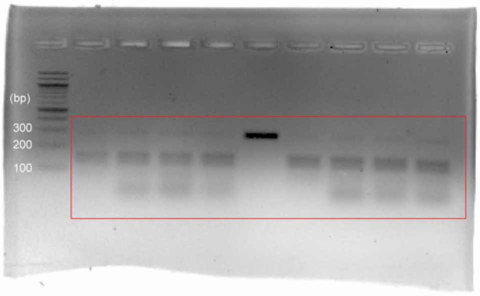

Supplementary Figure 3c

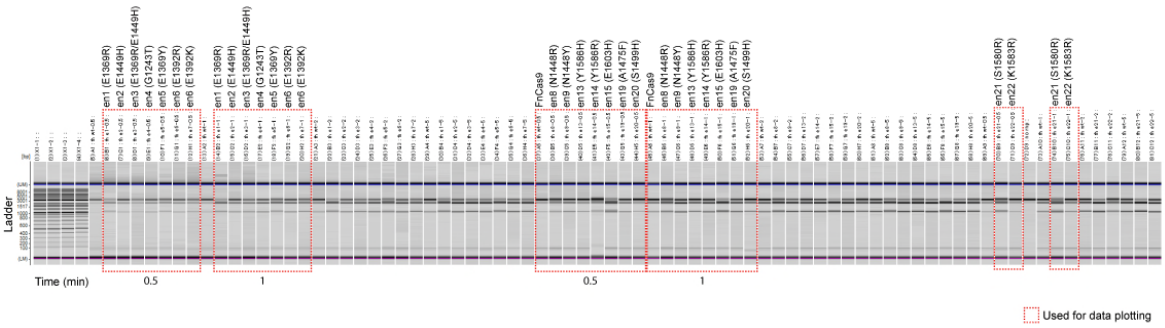

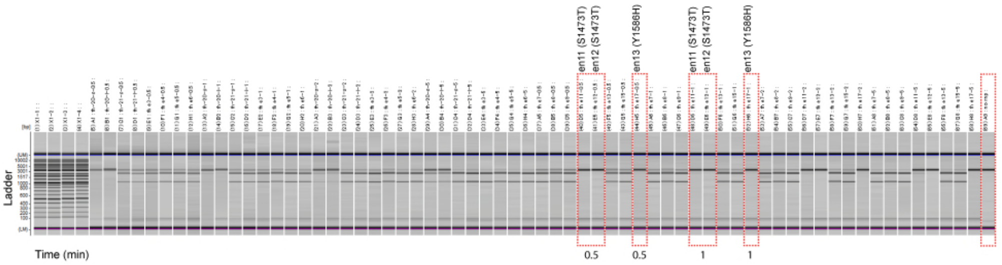

Supplementary Figure 3c

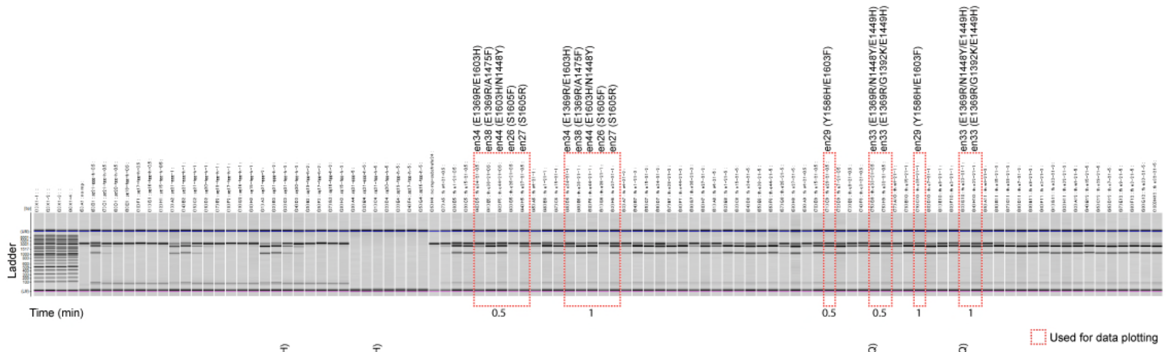

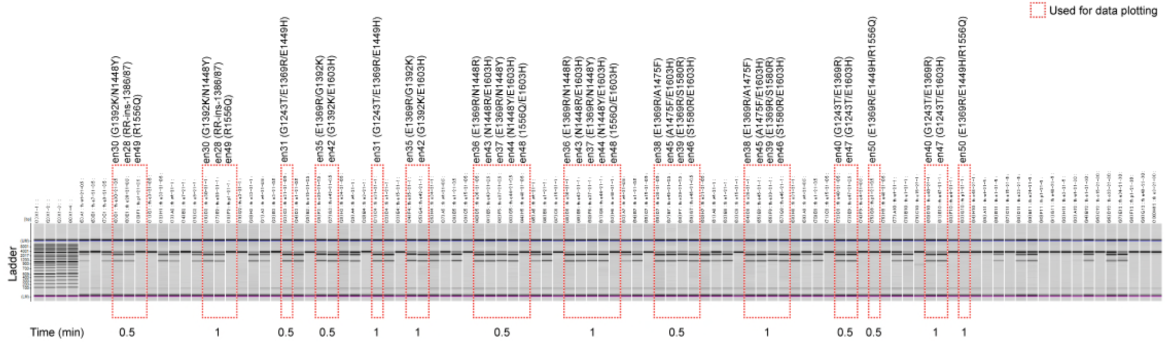

Supplementary Figure 3c

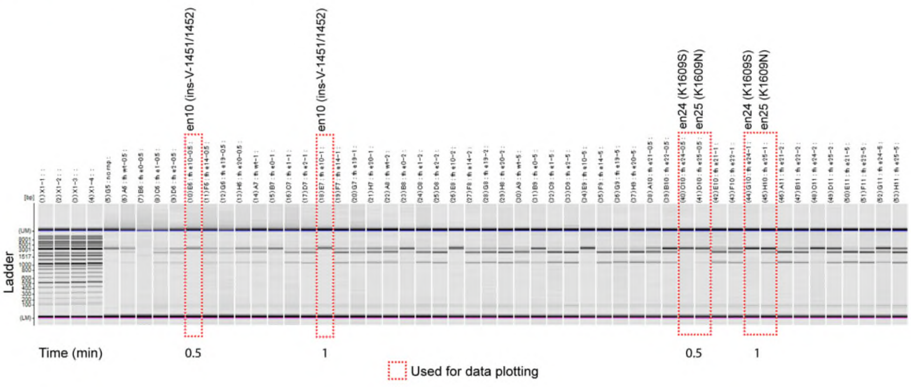

Source data: Uncropped gel for Supplementary Fig. 4a-d

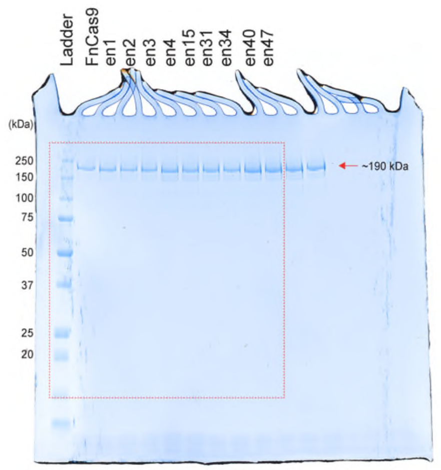

Supplementary Figure 4b

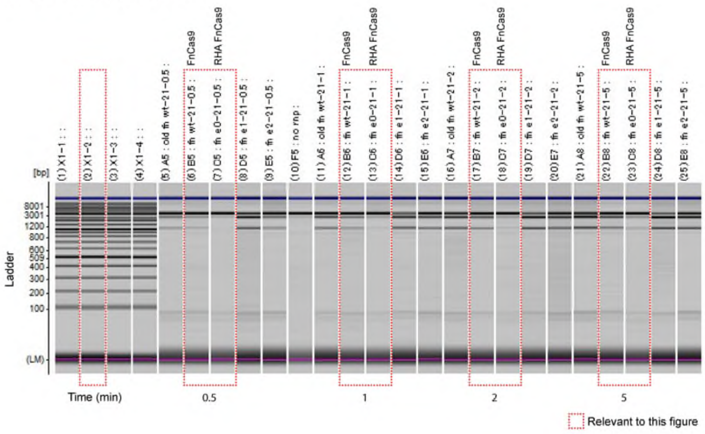

Supplementary Figure 4c

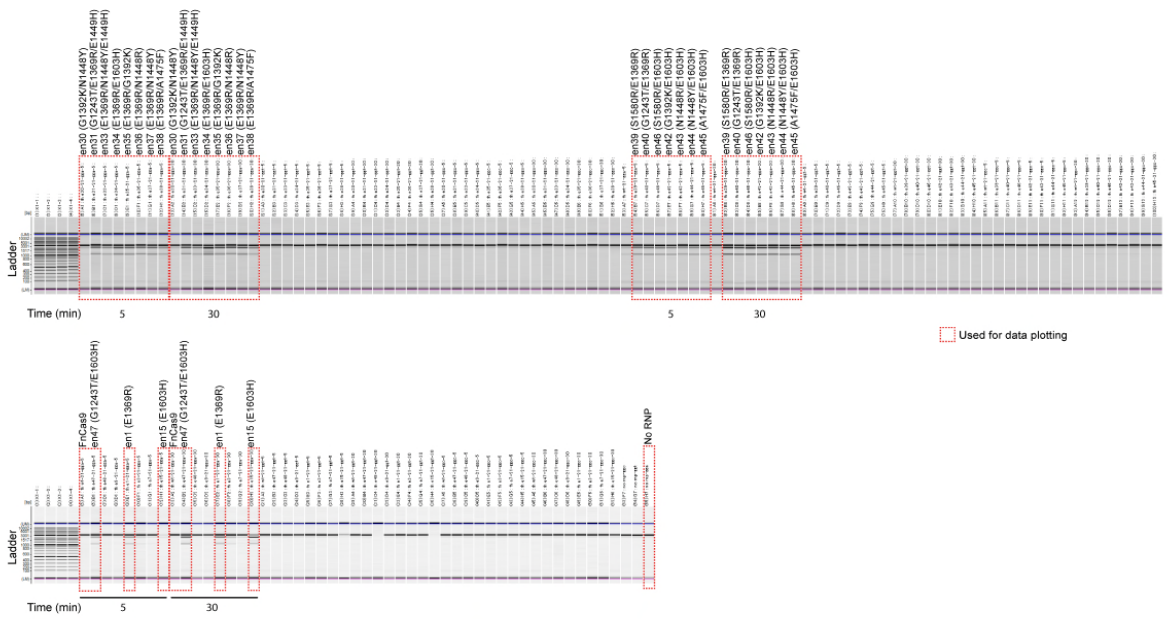

Supplementary Figure 4d

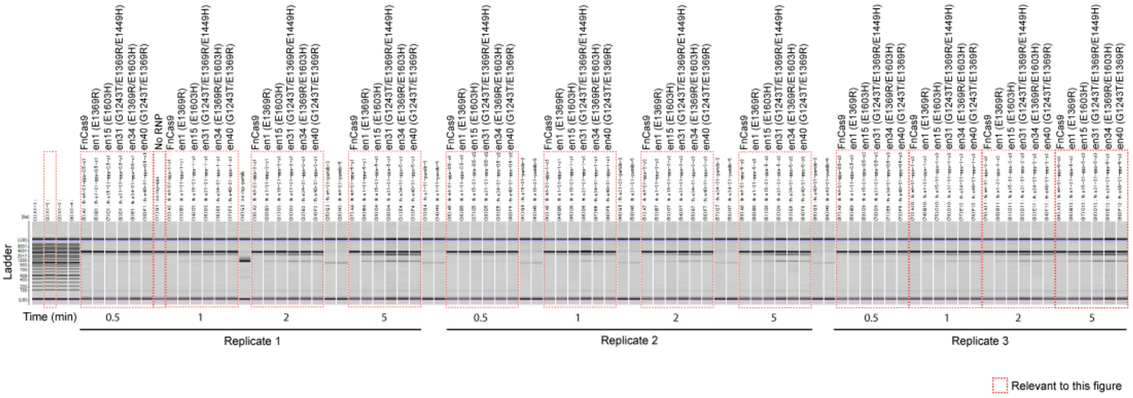

Source data: Uncropped gel for Supplementary Fig. 6b-d

Supplementary Figure 6b

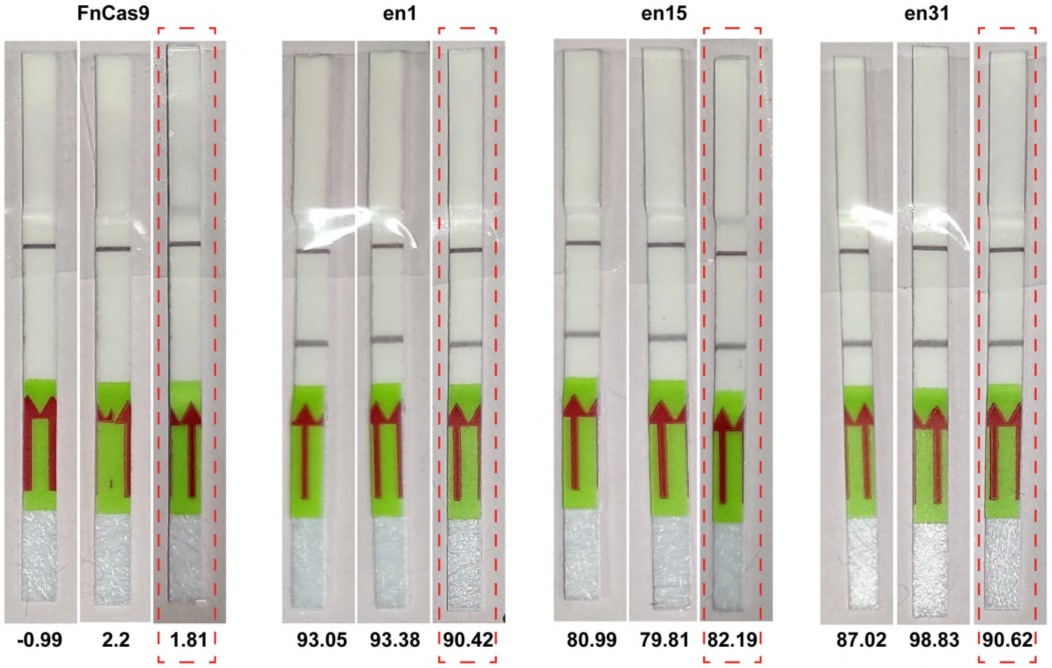

Supplementary Figure 6c

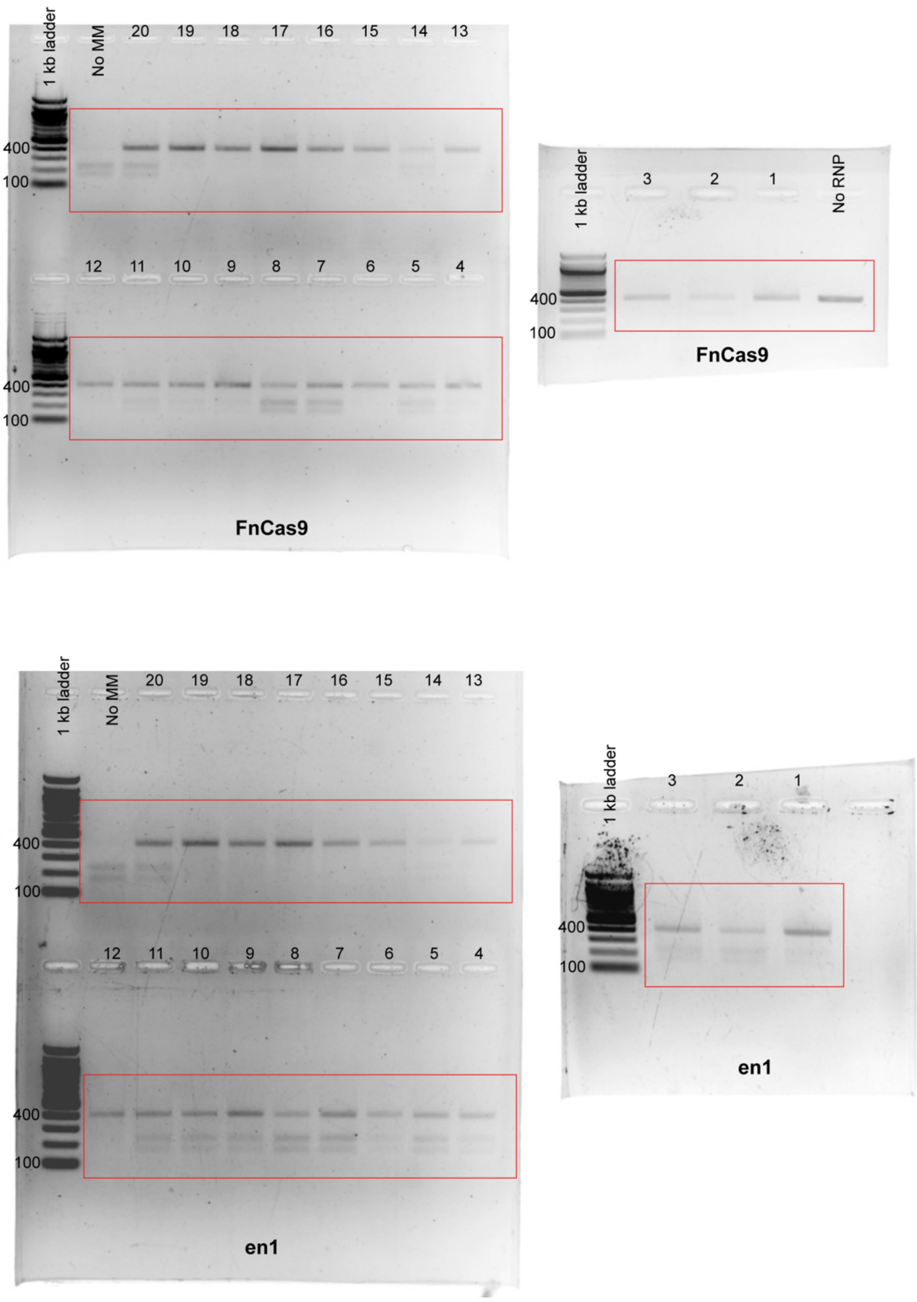

Supplementary Figure 6c

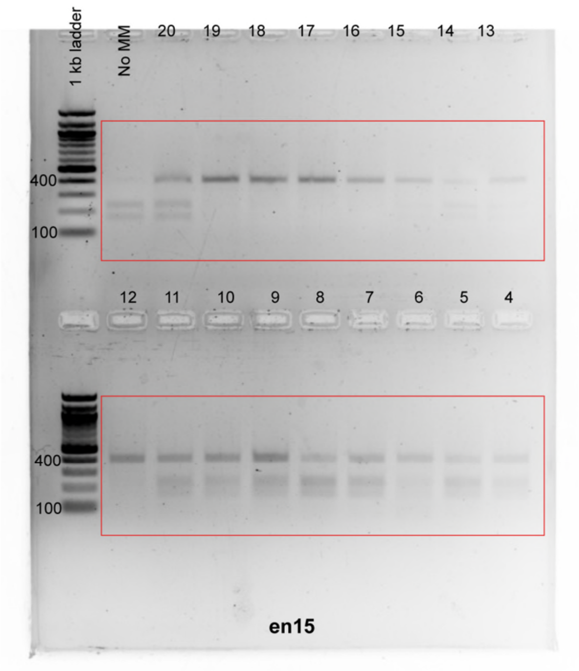

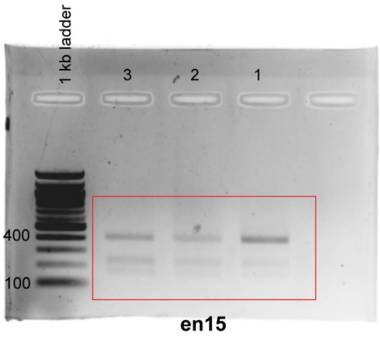

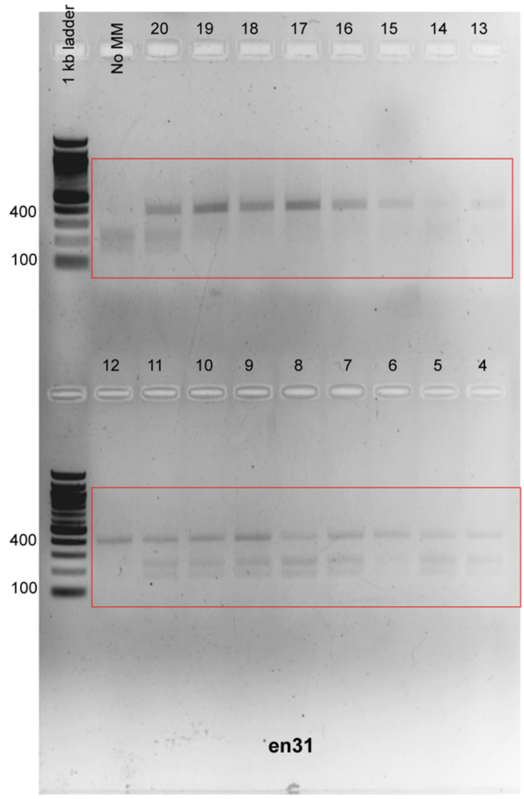

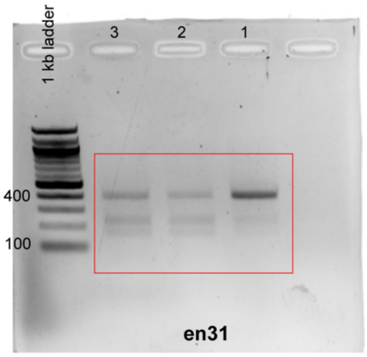

Supplementary Figure 6d

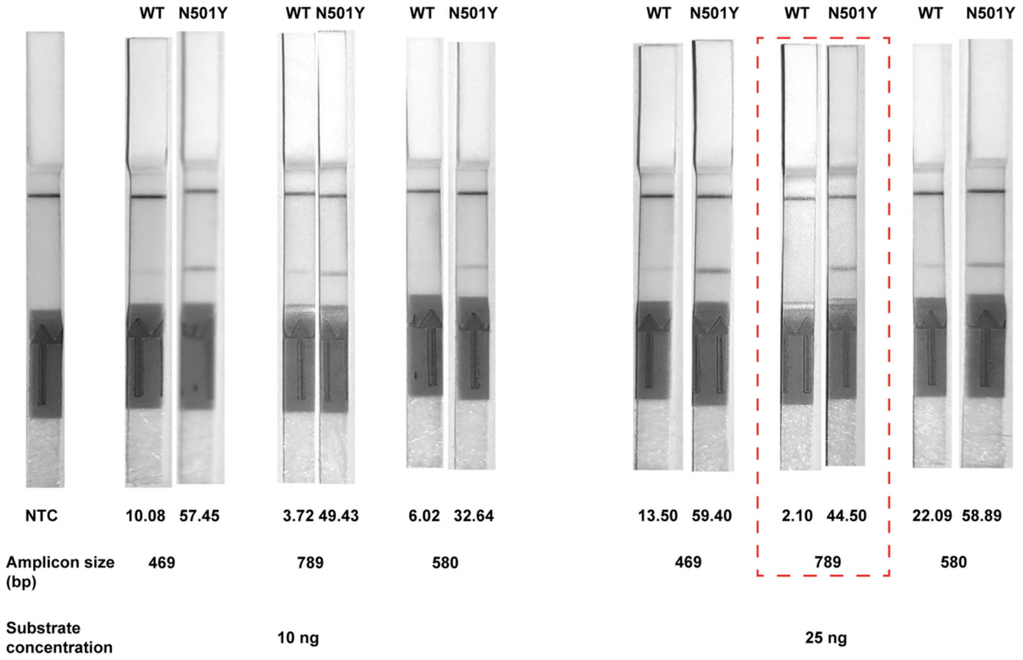

Source data: Uncropped gel for Supplementary Fig. 10h, i

Supplementary Figure 10h

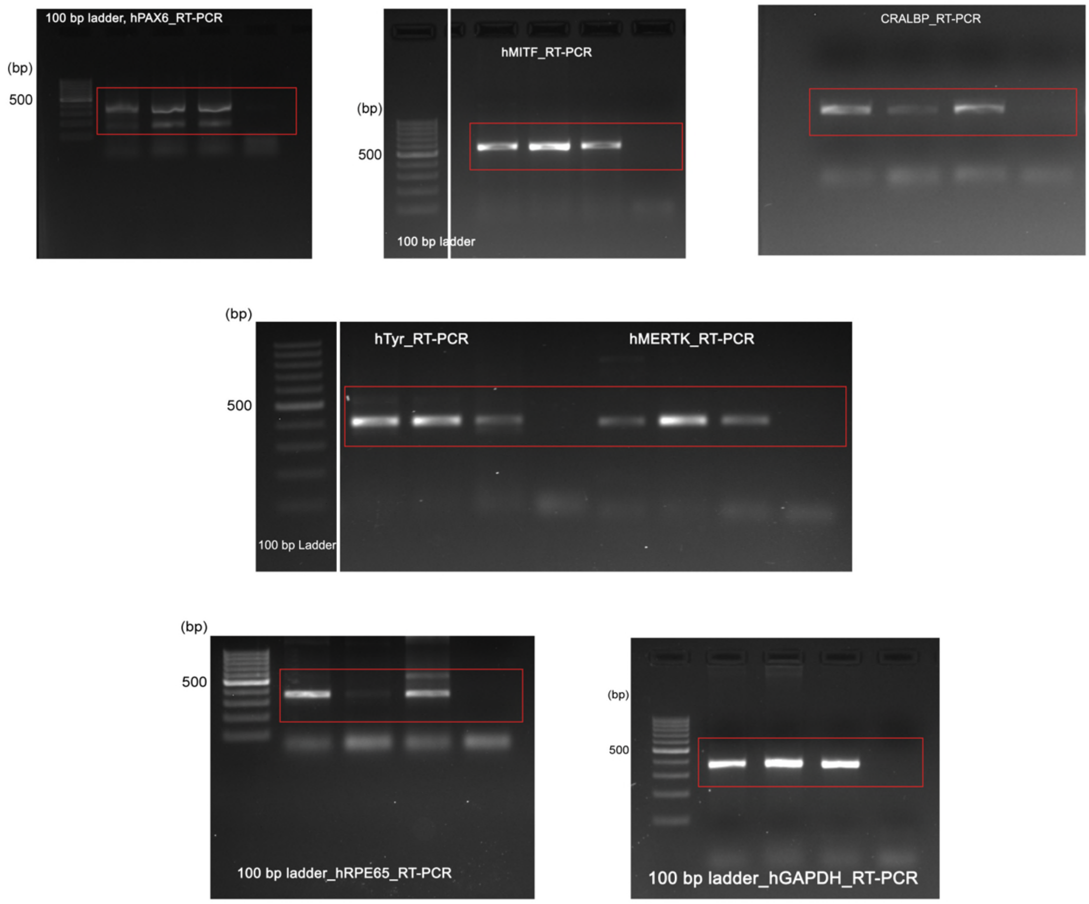

Supplementary Figure 10i

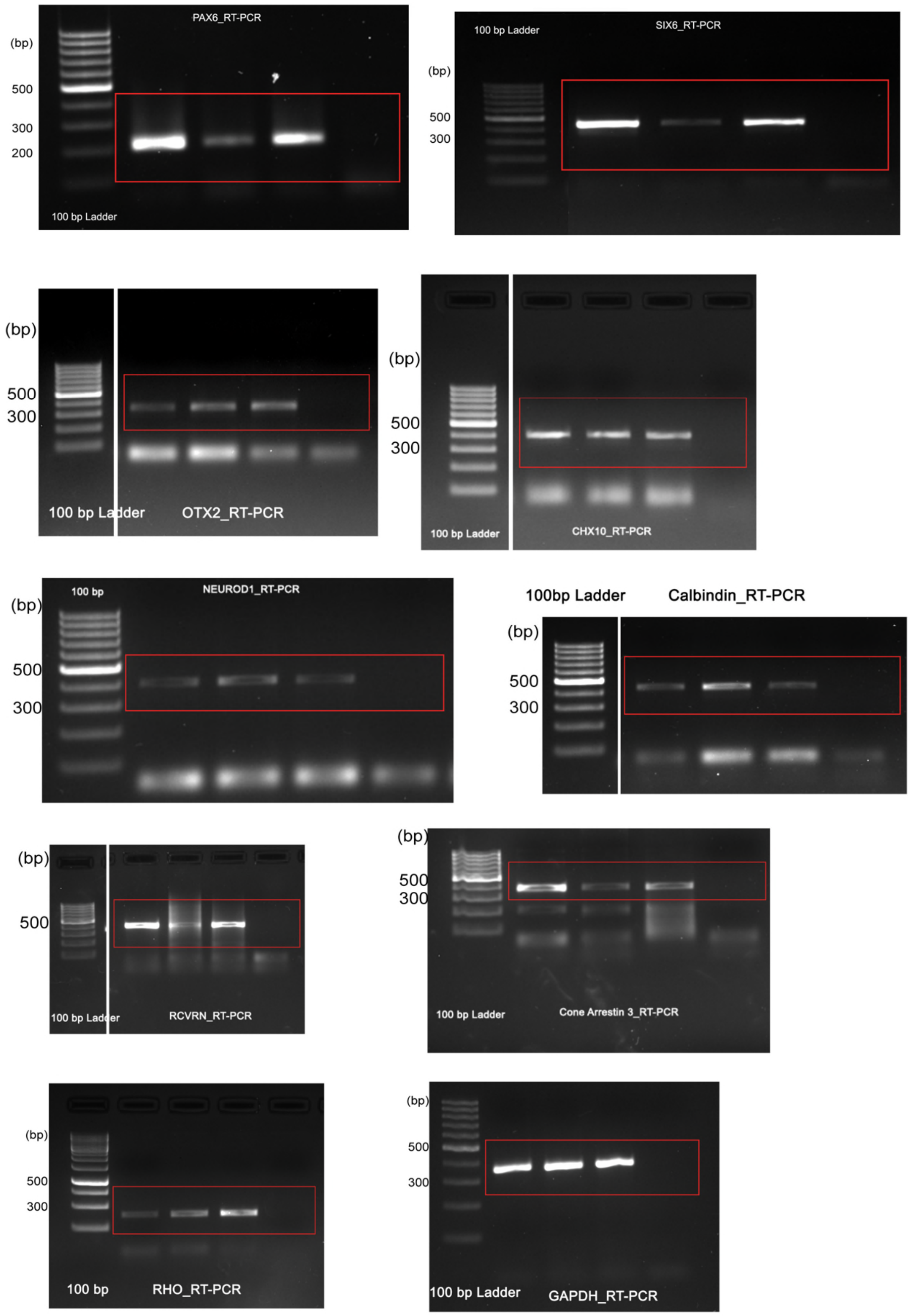

Source data: Uncropped gel for Supplementary Fig. 11c, d, f

Supplementary Figure 11c

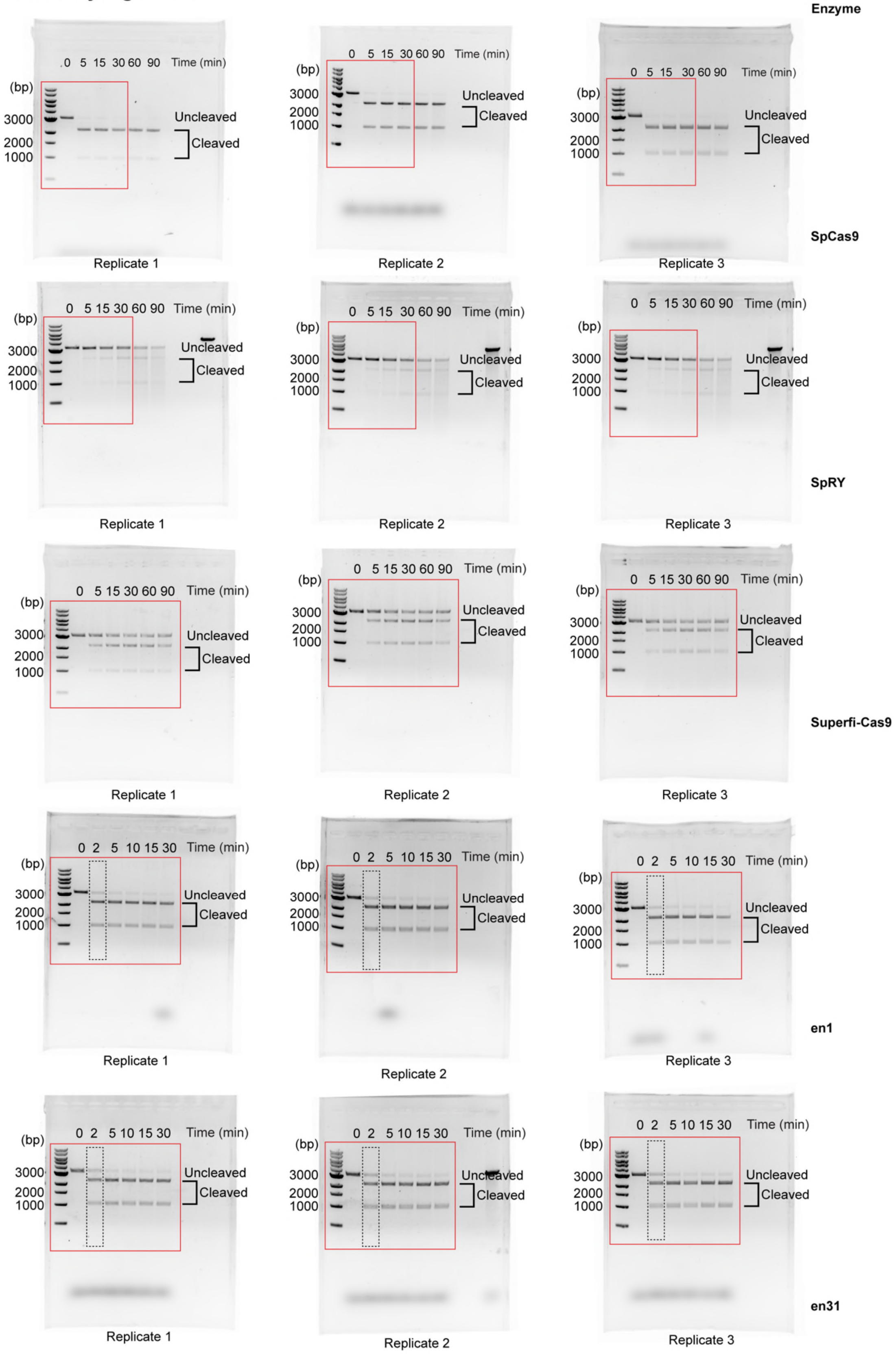

Supplementary Figure 11d

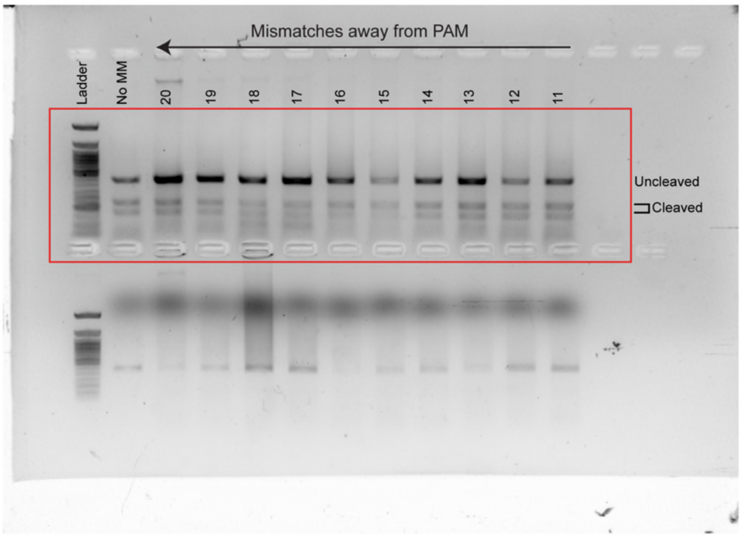

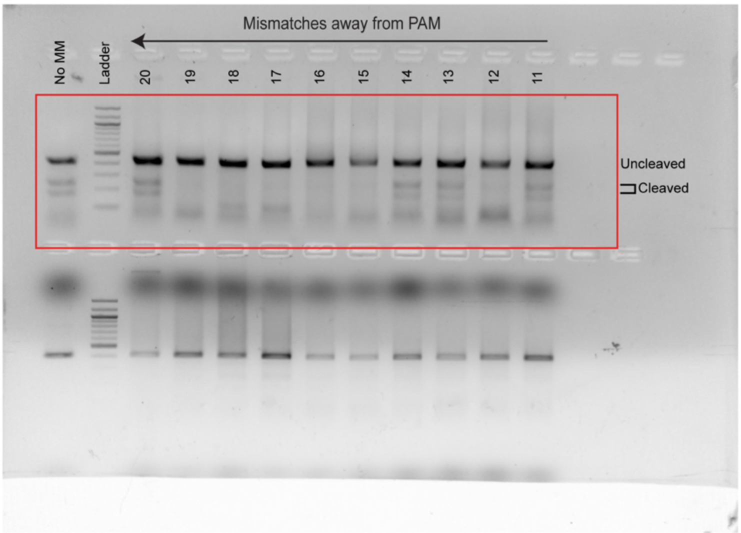

Supplementary Figure 11f

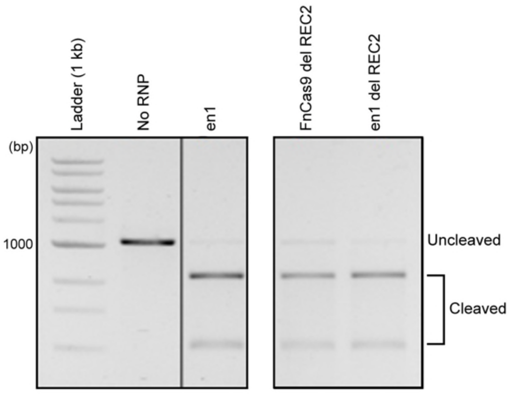

Supplementary Table 1: Construct details

Serial No.	Construct name	Addgene ID	Sequence Link	Reference
**1**	3xHA-dFnCas9-T2A-EGFP	201954	Link	This study
**2**	3xHA-dSpCas9-T2A-EGFP	201953	Link	This study
**3**	pE-SUMO-FnCas9	Not available	Link	Hirano *et al.* 2016
**4**	pUC119-PAMlib	Not available	Link	Nishimasu *et al.* 2018
**5**	pET-His6-dFnCas9GFP	130966	None	Acharya *et al.* 2019
**6**	pET-His6-en1dFnCas9GFP	201950	Link	This study
**7**	pET-His6-en15dFnCas9GFP	201951	Link	This study
**8**	PX458-3xHA-en1FnCas9	201947	Link	This study
**9**	PX458-3xHA-en15FnCas9	201948	Link	This study
**10**	PX458-3xHA-en31FnCas9	201949	Link	This study
**11**	PX458-3xHA-SpCas9	130968	None	Acharya *et al.* 2019
**12**	PX458-3xHA-SpCas9-NG	Not available	Link	This study
**13**	PX458-3xHA-SpRY		Link	This study
**14**	PX458-3xHA-FnCas9ABEmax8.17d	201955	Link	This study
**15**	PX458-3xHA-en31FnCas9ABEmax8.17d	201956	Link	This study
**16**	PX458-3xHA-SpNGABEmax8.17d	Not available	Link	This study
**17**	HDR donor-hDCX	Not available	Link	This study
