# Supplementary material for: PAM-flexible Engineered FnCas9 variants for robust and ultra-precise genome editing and diagnostics
Source: Nat Commun. 2024 Jun 28;15:5471. doi: 10.1038/s41467-024-49233-w (PMC11213958; doi:10.1038/s41467-024-49233-w)
Supplement: Supplementary file 6 — Description of Additional Supplementary Files [file 41467_2024_49233_MOESM6_ESM.pdf]

Description of Additional Supplementary Files

File Name: Supplementary Data 1

Description: PAM frequency table of Cas9 orthologs in the human genome used in mammalian cells

File Name: Supplementary Data 2

Description: Genomic coordinates from ChIP-seq assay.

1. dFn_myc_peaks_redundant;
2. dFn_myc_peaks_unique_NGG;

3.dSp_myc_peaks_redundant;

4. 4. dSp_myc_peaks_uniq_NGG

File Name: Supplementary Data 3

Description: Oligos used in the study

1. Oligos for plasmid constructions
2. T7 Oligos for IVT DNA template
3. Oligos for MST
4. Oligos for Mismatch walking assay
5. gRNA sequences for nuclease-based editing
6. gRNA sequences for base editing
7. Oligos for NGS
8. Oligos used for NGS analysis of PAM discovery assay
9. Oligos for HDR assay
10. Oligos for CRISPRDx
11. Oligos for Digenome-seq validation
12. Oligos for iPSC validation

File Name: Supplementary Data 4

Description: List of antibodies used in this study
