# Supplementary material for: PAM-flexible Engineered FnCas9 variants for robust and ultra-precise genome editing and diagnostics
Source: Nat Commun. 2024 Jun 28;15:5471. doi: 10.1038/s41467-024-49233-w (PMC11213958; doi:10.1038/s41467-024-49233-w)
Supplement: Supplementary file 8 — Reporting Summary [file 41467_2024_49233_MOESM8_ESM.pdf]

nature portfolio

Corresponding author(s): Sundaram Acharya and Debojyoti Chakraborty

Last updated by author(s): May 20, 2024

# Reporting Summary

Nature Portfolio wishes to improve the reproducibility of the work that we publish. This form provides structure for consistency and transparency in reporting. For further information on Nature Portfolio policies, see our Editorial Policies and the Editorial Policy Checklist.

## Statistics

For all statistical analyses, confirm that the following items are present in the figure legend, table legend, main text, or Methods section.

| n/a | Confirmed | |
|---|---|---|
| ☐ | ☒ | The exact sample size (*n*) for each experimental group/condition, given as a discrete number and unit of measurement |
| ☐ | ☒ | A statement on whether measurements were taken from distinct samples or whether the same sample was measured repeatedly |
| ☐ | ☒ | The statistical test(s) used AND whether they are one- or two-sided<br>*Only common tests should be described solely by name; describe more complex techniques in the Methods section.* |
| ☒ | ☐ | A description of all covariates tested |
| ☒ | ☐ | A description of any assumptions or corrections, such as tests of normality and adjustment for multiple comparisons |
| ☐ | ☒ | A full description of the statistical parameters including central tendency (e.g. means) or other basic estimates (e.g. regression coefficient) AND variation (e.g. standard deviation) or associated estimates of uncertainty (e.g. confidence intervals) |
| ☐ | ☒ | For null hypothesis testing, the test statistic (e.g. *F*, *t*, *r*) with confidence intervals, effect sizes, degrees of freedom and *P* value noted<br>*Give P values as exact values whenever suitable.* |
| ☒ | ☐ | For Bayesian analysis, information on the choice of priors and Markov chain Monte Carlo settings |
| ☒ | ☐ | For hierarchical and complex designs, identification of the appropriate level for tests and full reporting of outcomes |
| ☒ | ☐ | Estimates of effect sizes (e.g. Cohen's *d*, Pearson's *r*), indicating how they were calculated |

*Our web collection on statistics for biologists contains articles on many of the points above.*

## Software and code

Policy information about availability of computer code

| | |
|---|---|
| Data collection | Sanger sequencing data were obtained as ab1 files. Capillary electrophoresis data were automated and obtained as CSV files. Agarose gel electrophoresis data were obtained using ImageJ. Next generation sequencing data were obtained as FASTQ files after sequencing on either MiniSeq platform (amplicon sequencing) or MiSeq platform (PAM discovery) or HiSeq X platform (ChIP-SEQ) or NovaSeq 6000 platform. |
| Data analysis | Bowtie 2, MACS2, bedtools, Intervene, CRISPResso2 v2.0.45 for indel and base editing analysis. Python 3.7 for PAM frequency analysis. R 4.2 and GraphPad Prism 8 for data visualization. COOT for protein structure analysis and CueMol (v2.2.3.443) for structure visualization. OriginLab for binding data. KronaTools (v2.7) for PAM wheel and WebLogo3 for sequence logo representations. FastQC, MultiQC, Cutadapt, Samtools for analysis of Digenome-seq data analysis. Circos and UpsetR for Digenome-seq data visualization. All relevant links and details can be found in the manuscript. |

For manuscripts utilizing custom algorithms or software that are central to the research but not yet described in published literature, software must be made available to editors and reviewers. We strongly encourage code deposition in a community repository (e.g. GitHub). See the Nature Portfolio guidelines for submitting code & software for further information.

## Data

Policy information about availability of data

All manuscripts must include a data availability statement. This statement should provide the following information, where applicable:

- Accession codes, unique identifiers, or web links for publicly available datasets
- A description of any restrictions on data availability
- For clinical datasets or third party data, please ensure that the statement adheres to our policy

A subset of selected plasmids encoding relevant constructs used in this study will be available through Addgene (Deposit ID 82698). Other plasmids can be made available upon request. Deep sequencing data from ChIP, amplicon sequencing and Digenome-seq experiments were deposited as a BioProject under the Project ID PRJNA766155 and will be made available from the NCBI sequence read archive (SRA). The iPSC lines registered at hPSCreg (see 'cell line source' for details).

## Research involving human participants, their data, or biological material

Policy information about studies with human participants or human data. See also policy information about sex, gender (identity/presentation), and sexual orientation and race, ethnicity and racism.

| | |
|---|---|
| Reporting on sex and gender | Patient line & Edited line: Male, Normal control line: Female |
| Reporting on race, ethnicity, or other socially relevant groupings | Indian |
| Population characteristics | See above |
| Recruitment | The participants were recruited as a part of a completed IRB/IEC approved study on "the genetic screening of RP & LCA patients for the identification of pathogenic mutations". Probands with confirmed mutations in RPE65 gene were counselled for their participation in an independent IRB/IEC approved study on "Generation of induced pluripotent stem cells (iPSCs) from human dermal fibroblasts (HDFs) or peripheral blood mononuclear cells (PBMCs) of patients with inherited eye diseases". Volunteered participants were further counselled and informed consents taken before collecting their skin and blood samples for experimentation. We declare no selection bias that can potentially bias the results reported. |
| Ethics oversight | The study was approved by the Institutional Ethics Committee (IEC), Institutional Bio-Safety Committee (IBSC) and Institutional Committee for Stem Cell Research (IC-SCR) at the LV Prasad Eye Institute, Hyderabad. |

Note that full information on the approval of the study protocol must also be provided in the manuscript.

# Field-specific reporting

Please select the one below that is the best fit for your research. If you are not sure, read the appropriate sections before making your selection.

[x] Life sciences [ ] Behavioural & social sciences [ ] Ecological, evolutionary & environmental sciences

For a reference copy of the document with all sections, see nature.com/documents/nr-reporting-summary-flat.pdf

# Life sciences study design

All studies must disclose on these points even when the disclosure is negative.

| | |
|---|---|
| Sample size | Triplicate or more biological samples were evaluated in all assays unless stated otherwise in the figure legends of the manuscript. Sample size calculation was not performed. |
| Data exclusions | No data was excluded from analysis. |
| Replication | All attempts at replication was successful. |
| Randomization | Randomization is not relevant to this study. |
| Blinding | Blinding is not relevant to this study. |

# Reporting for specific materials, systems and methods

We require information from authors about some types of materials, experimental systems and methods used in many studies. Here, indicate whether each material, system or method listed is relevant to your study. If you are not sure if a list item applies to your research, read the appropriate section before selecting a response.

## Materials & experimental systems

| n/a | Involved in the study | |
|---|---|---|
| ☐ | ☒ | Antibodies |
| ☐ | ☒ | Eukaryotic cell lines |
| ☒ | ☐ | Palaeontology and archaeology |
| ☒ | ☐ | Animals and other organisms |
| ☒ | ☐ | Clinical data |
| ☒ | ☐ | Dual use research of concern |
| ☒ | ☐ | Plants |

## Methods

| n/a | Involved in the study | |
|---|---|---|
| ☐ | ☒ | ChIP-seq |
| ☐ | ☒ | Flow cytometry |
| ☒ | ☐ | MRI-based neuroimaging |

# Antibodies

| | |
|---|---|
| Antibodies used | Anti-HA, ChIP grade (abcam, ab9110), Anti-PAX6 (Abcam, Cat No. ab195045), Anti-HA (CST, Cat No. 3724S), Anti-OCT-4 [POU5F1], clone: 7F9.2 (Cat No. MAB4419), Anti-NANOG clone 7F7.1 (Cat No. MABD24), Anti-Stage-Specific Embryonic Antigen-4, clone MC-813-70 (Cat No. MAB4304), anti-SOX2 (Abcam, Cat No. ab97959), Recombinant Anti-KLF4 [EPR19590] (Cat No. ab215036), Alexa Fluor™ 488 Phalloidin (Invitrogen, Thermo Fisher Scientific, A12379), Anti-MiTF [D5], Mouse mAb (abcam, ab3201), Anti-RPE65 [EPR22579-44], Rabbit mAb (abcam, ab231782), Anti-RPE65, Rabbit Polyclonal (Thermo Scientific, PA5-115852), Anti-CRALBP, Mouse mAb (abcam, ab15051), Anti-beta-actin (abcam, ab8227). |
| Validation | Antibodies were validated by the indicated manufacturer before purchase. |

# Eukaryotic cell lines

Policy information about cell lines and Sex and Gender in Research

| | |
|---|---|
| Cell line source(s) | HEK293T-ATCC-CRL-3216, ARPE-19-ATCC-CRL-2302, LVPEIi001-B (LVIP02-NC-F2-1), LVPEIi005-A (LVIP02-LC2-2), LVPEIi005-A-1 (LVIP02-LC2-2-BE1) |
| Authentication | All the cell lines were authenticated before conducting gene editing experiments. |
| Mycoplasma contamination | Cell lines were tested negative for mycoplasma contamination. |
| Commonly misidentified lines (See ICLAC register) | None used in this study. |

# Plants

| | |
|---|---|
| Seed stocks | *Report on the source of all seed stocks or other plant material used. If applicable, state the seed stock centre and catalogue number. If plant specimens were collected from the field, describe the collection location, date and sampling procedures.* |
| Novel plant genotypes | *Describe the methods by which all novel plant genotypes were produced. This includes those generated by transgenic approaches, gene editing, chemical/radiation-based mutagenesis and hybridization. For transgenic lines, describe the transformation method, the number of independent lines analyzed and the generation upon which experiments were performed. For gene-edited lines, describe the editor used, the endogenous sequence targeted for editing, the targeting guide RNA sequence (if applicable) and how the editor was applied.* |
| Authentication | *Describe any authentication procedures for each seed stock used or novel genotype generated. Describe any experiments used to assess the effect of a mutation and, where applicable, how potential secondary effects (e.g. second site T-DNA insertions, mosiacism, off-target gene editing) were examined.* |

# ChIP-seq

## Data deposition

☒ Confirm that both raw and final processed data have been deposited in a public database such as GEO.

☒ Confirm that you have deposited or provided access to graph files (e.g. BED files) for the called peaks.

| | |
|---|---|
| Data access links *May remain private before publication.* | *For "Initial submission" or "Revised version" documents, provide reviewer access links. For your "Final submission" document, provide a link to the deposited data.* |
| Files in database submission | *Provide a list of all files available in the database submission.* |
| Genome browser session (e.g. UCSC) | *Provide a link to an anonymized genome browser session for "Initial submission" and "Revised version" documents only, to enable peer review. Write "no longer applicable" for "Final submission" documents.* |

## Methodology

| | |
|---|---|
| Replicates | One replicate each for dSpCas9 and dFnCas9 |
| Sequencing depth | dFn_Myc 24663290 reads, 151 read length, pair-end<br>dSp_Myc 28821619 reads, 151 read length, pair-end<br>dSp_Input 20863898 reads, 151 read length, pair-end<br>dFn_Input 19790433 reads, 151 read length, pair-end |
| Antibodies | Anti-HA, ChIP grade (abcam, ab9110) |
| Peak calling parameters | Raw data quality is checked with FastQC. Reads with poor quality was filtered using trimmomatic by applying sliding window of 4:15 (keeping a threshold of 15 for continuous four bases). Raw sequencing reads were mapped to the human reference genome GRCh38 using bowtie. Peaks were called over input samples using MACS278 with default parameters. Finally, scrambled sample peaks were used to remove background and false positive peaks from the dSpCas9 and dFnCas9 test samples. These filtered peaks were searched for off-targets based on sgRNA sequence homology with a maximum of 6 mismatches. On-target peak coverage plots were generated by the fluff profiles command with 'remove duplicates' option. Overlap between the dSpCas9 and dFnCas9 ChIP peaks were calculated using bedtools and plotted as weighted Venn diagrams with the help of Intervene. |
| Data quality | See Supplementary Table 3 |
| Software | Bowtie, MACS278, bedtools, Intervene |

# Flow Cytometry

## Plots

Confirm that:

- [x] The axis labels state the marker and fluorochrome used (e.g. CD4-FITC).
- [x] The axis scales are clearly visible. Include numbers along axes only for bottom left plot of group (a 'group' is an analysis of identical markers).
- [ ] All plots are contour plots with outliers or pseudocolor plots.
- [x] A numerical value for number of cells or percentage (with statistics) is provided.

## Methodology

| | |
|---|---|
| Sample preparation | Mammalian cells transfected with relevant plasmids were harvested in PBS for analysis |
| Instrument | BD FACSMelody™ Cell Sorter |
| Software | BD FACSChorus™ Software for collection of data. FlowJo v10 for data analysis. |
| Cell population abundance | 10,000 gated events for data analysis were collected based on default FSC/SSC parameters. |
| Gating strategy | The GFP+ gating was done by subtracting the FITC-A readings of Cas9-T2A-EGFP plasmid transfected cells from untransfected control cells. |

- [x] Tick this box to confirm that a figure exemplifying the gating strategy is provided in the Supplementary Information.
